# An MR spectroscopy study of temporal areas excluding primary auditory cortex and frontal regions in subjective bilateral and unilateral tinnitus

**DOI:** 10.1038/s41598-023-45024-3

**Published:** 2023-10-27

**Authors:** Joanna Wójcik, Bartosz Kochański, Katarzyna Cieśla, Monika Lewandowska, Lucyna Karpiesz, Iwona Niedziałek, Danuta Raj-Koziak, Piotr Henryk Skarżyński, Tomasz Wolak

**Affiliations:** 1 Bioimaging Research Center, World Hearing Center, Institute of Physiology and Pathology of Hearing, Mokra 17 Street, Kajetany, 05-830 Nadarzyn, Poland; 2 Faculty of Philosophy and Social Sciences, Institute of Psychology, Nicolaus Copernicus University, Fosa Staromiejska 1a Street, 87-100 Toruń, Poland; 3 Tinnitus Department, World Hearing Center, Institute of Physiology and Pathology of Hearing, Mokra 17 Street, Kajetany, 05-830 Nadarzyn, Poland; 4 Department of Teleaudiology and Screening, World Hearing Center, Institute of Physiology and Pathology of Hearing, Mokra 17 Street, Kajetany, 05-830 Nadarzyn, Poland; 5 Institute of Sensory Organs, Mokra 1 Street, Kajetany, 05-830 Nadarzyn, Poland; 6 Heart Failure and Cardiac Rehabilitation Department, Faculty of Medicine, Medical University of Warsaw, Kondratowicza 8 Street, 03-242 Warsaw, Poland

**Keywords:** Biochemistry, Neuroscience, Psychology, Medical research

## Abstract

Previous studies indicate changes in neurotransmission along the auditory pathway in subjective tinnitus. Most authors, however, investigated brain regions including the primary auditory cortex, whose physiology can be affected by concurrent hearing deficits. In the present MR spectroscopy study we assumed increased levels of glutamate and glutamine (Glx), and other Central Nervous System metabolites in the temporal lobe outside the primary auditory cortex, in a region involved in conscious auditory perception and memory. We studied 52 participants with unilateral (n = 24) and bilateral (n = 28) tinnitus, and a control group without tinnitus (n = 25), all with no severe hearing losses and a similar hearing profile. None of the metabolite levels in the temporal regions of interest were found related to tinnitus status or laterality. Unexpectedly, we found a tendency of increased concentration of Glx in the control left medial frontal region in bilateral vs unilateral tinnitus. Slightly elevated depressive and anxiety symptoms were also shown in participants with tinnitus, as compared to healthy individuals, with the bilateral tinnitus group marginally more affected. We discuss no apparent effect in the temporal lobes, as well as the role of frontal brain areas, with respect to hearing loss, attention and psychological well-being in chronic tinnitus. We furthermore elaborate on the design-related and technical obstacles of MR spectroscopy.

## Introduction

Subjective tinnitus is an auditory percept (such as e.g. buzzing, ringing, humming), experienced despite absence of any identifiable external sound sources. It can be perceived as unilateral (one-sided), bilateral (in both ears or in the head) or with changing laterality. Most authors suggest primary peripheral auditory damage as the main trigger for tinnitus occurrence, through the mechanism of disruption/deafferentation of the neuronal activity^1–7^. This initial trigger is believed to then spark a series of further changes up along the auditory pathway, including in the spontaneous and/or sound-induced neural activity in regions spanning from the auditory nerve to the primary auditory cortical areas, as shown in animal models of tinnitus^3,4,8,9^ and in humans^10–15^.

A critical challenge when studying neurotransmission (and metabolism) in the auditory pathway, including in the primary auditory cortex, is that changes due to tinnitus cannot be easily disentangled from those resulting from a hearing loss. This especially concerns animal models, with tinnitus induced by noise, physical or chemical trauma, thereby surely leading to severe hearing deficits^16–18^, but also studies in humans with the participants often representing various levels of hearing loss^10–15^.

At the molecular level, tinnitus is described in the framework of disrupted excitation-inhibition homeostasis, mainly mediated by glutamate (Glu) and gamma-aminobutyric acid (GABA), at several levels of the auditory pathway, including in the primary auditory cortex^4,19^. Invasive studies in rodents with behavioral signs of tinnitus have shown changes in both these neurotransmitter systems, such as altered concentrations, tissue distribution, receptor affinity and density, as well as transporter function^16,20–25^. These animal findings led to initiating several drug trials for tinnitus treatment, including of glutamate antagonists^26,27^. Nevertheless, with most of these trials discontinued, it has been emphasized that much more research is still required regarding the pathophysiology of tinnitus, especially that the animal model of tinnitus and tinnitus in humans might involve different mechanisms^28^.

Metabolite and neurotransmitter concentrations in the central nervous system can be studied using the non-invasive technique of ^1^H-MRS (proton magnetic resonance spectroscopy, hereafter MRS)^29^. In fact, Brozoski and colleagues^30^ were one of the first to apply MRS in an animal model of tinnitus, and showed bidirectional changes in GABA and glutamate levels in the cochlear nucleus, medial geniculate body and the primary auditory cortex of rats with behavioral signs of tinnitus (and hearing loss). The method has also been proposed as a tool to study molecular aspects of tinnitus in humans^31^. Up until now, however, its implementation has been very limited.

To the best of our knowledge, there have been only three works investigating Glu or GABA levels in tinnitus, with the authors mainly focused on the primary auditory cortex, as most probably inspired by animal models^19,32,33^. These works also included participants with various hearing levels and mixed tinnitus lateralities, which might have contributed to the inconsistent results. At the same time, literature exploring the reasons for developing uni- or bilateral tinnitus is extremely scarce, with only single authors discussing hearing level asymmetries^34^, genetic factors^35^, and involvement of higher order brain areas^36^, warranting further research.

To address the several raised issues, in the current MRS work in > 50 people with tinnitus, we set to investigate metabolite levels in temporal brain areas excluding the primary auditory cortex, while taking into account hearing levels of the participants and the perceived tinnitus laterality.

Specifically, we used single-voxel MRS and an optimized clinical sequence to measure glutamate/glutamine (Glx) composite concentration as a proxy for Glu. We chose two regions of interest in bilateral superior/medial temporal areas. Two reference regions in the left and right medial frontal areas were also examined, in order to test the stability of the MRS measurements. The locations were selected individually for each person, in areas remote from bone, blood vessels and air tissue, in order to obtain high-quality MRS signals. Brain tissue segmentation was performed to ensure the same content of white and gray matter across groups^37–39^.

The specific temporal region of interest has been shown to be involved in conscious auditory perception and auditory memory^6,40–42^^.^ It was also selected with the hope to minimize the potential confounding effect of hearing levels on metabolite concentrations. Even though all participants of the study had normal hearing, except for several with a more severe hearing loss in middle/high frequency ranges, there is evidence that tinnitus can stem from even a minor or a “hidden” hearing deficit undetectable with conventional pure tone audiometry^6,36,43–49^.

In order to address the issue of tinnitus laterality^46^, we also applied a clear distinction between a subgroup of participants that experienced tinnitus as bilateral and those whose tinnitus was perceived as unilateral.

In addition, with a number of behavioral studies showing a bidirectional relationship between tinnitus and psychological challenges, such as distress and affective disorders^50–54^, participants with affective disorders, such as depression or anxiety, were excluded from the study.

As our main hypothesis, we expected to see, (a) increased glutamate levels (indicating increased neuronal excitation) in participants with tinnitus as compared to those with no tinnitus, (b) a relationship between glutamate levels and tinnitus laterality.

As part of general exploration, levels of several other metabolites were also measured, i.e. N-acetylaspartate (NAA), choline (Cho) and myoinositol (mI), all typically assessed in clinical brain MRS, pertaining to their different functions in maintaining a healthy central nervous system (CNS), including in the auditory system^19,29,55–57^.

## Material and methods

### Participants

#### General demographics and exclusion criteria

Seventy seven (77) participants in total were included in the study, with 25 healthy subjects (group C) and 52 patients with tinnitus (24 with unilateral tinnitus, group TU, and 28 with bilateral tinnitus, group TB). Screening included an interview, a detailed medical examination of the ear and an anatomical MRI T1 head scan, as well as a set of questionnaires. Participants with the following impairments or medical history were excluded from the study: somatic tinnitus, acoustic tumors, brain tumors, stroke, neurovascular conflict, Meniere’s disease, cerebellopontine angle tumors, tumors of the internal meatus, meningioma, astrocytoma, MELAS syndrome, history of surgeries of the auditory pathway or the brain, history of neurological or psychiatric disorders (including clinical depression). The demographic data of the participants is presented in Table 1.

**Table 1:** Demographic information.

Characteristic^a^	C, N = 25^b^	TU, N = 24^b^	TB, N = 28^b^	*p*-value^c^
Age				0.7
Median (IQR)	42 (38–51)	48 (38–56)	44 (37–52)	
Mean (SD)	45 (10)	46 (13)	44 (11)	
Range	30–71	21–69	24–61	
Sex				*0.059*
Female	10/25 (40%)	16/24 (67%)	10/28 (36%)	
Male	15/25 (60%)	8/24 (33%)	18/28 (64%)	

^a^SD—standard deviation, IQR—inter-quartile interval.

^b^C—control, TU—tinnitus unilateral, TB—tinnitus bilateral For sex: n/N (%); n—subgroup count, N—whole group count;

^c^For age: Kruskal–Wallis rank sum test; For sex: Pearson’s Chi-squared test.

*p* < 0.1 are in italics.

#### The characteristics of tinnitus

The included fifty-two (52) participants with subjective tinnitus had been recruited from among the patients of the Institute of Physiology and Pathology of Hearing. All had experienced tinnitus for at least 6 months continually (considered *chronic* tinnitus). Unilateral tinnitus was described by most participants as “I hear it on the left/right side” (20 out of 24 perceived their tinnitus as left-lateralized and 4 as right-lateralized). Five out of 24 participants presented clear one-ear dominance, while perceiving minor tinnitus in the other ear. Bilateral tinnitus was described by the participants as “I hear it in the head” or “I hear it in both ears”. Five out of 28 people reported that their tinnitus laterality was changing, but generally affecting both ears. Subjective experience of tinnitus was variable among the participants, and they characterized the sound as tonal, hissing, buzzing or ringing, as well constant or changing in time, frequency and/or intensity. As potential factors contributing to the occurrence of their tinnitus, 16 (31%) participants mentioned noise exposure, 12 (23%) mentioned stress, while 17 (33%) could not name any such factor. The remaining 7 (13%) linked their tinnitus to factors such as injury, infection, inflammation, vertigo or a combination of various factors (e.g. stress and noise). The response distribution was similar in the two subgroups. None of the participants took part in any kind of therapy or training targeted at tinnitus prior to or during the current study.

Participants with tinnitus completed the following questionnaires referring to their tinnitus experience:

1. *Tinnitus Handicap Inventory (THI)* consisting of 25 questions describing the influence of tinnitus on everyday life. The scoring scale is from 0 to 100 points. The score 0–16 is interpreted as slight influence of tinnitus, 18–36 as mild, 38–56 as moderate, 58–76 as severe, and 78–100 as catastrophic influence^58–60^.
2. *Tinnitus Functional Index (TFI)* consisting of 25 questions describing how the respondent has been feeling during the last week, with respect to tinnitus. The general score of TFI is on a scale of 0–100 points. Tinnitus is considered a small problem when the scores are in the range between 0 and 18 points. For higher scores, the following interpretation is used: 18–42 points—a moderate problem, 42–65 points—a significant problem, 65–100 points—a very significant problem^60–62^.
3. *The Polish Tinnitus Characteristics Inventory* (the original Polish name: “Kwestionariusz charakterystyki szumów usznych”) which is an in-house instrument developed by specialists of the Institute of Physiology and Pathology of Hearing. The inventory measures, among others, on a VAS scale, persistence of tinnitus (“Please, rate the persistence of your tinnitus on a scale from 1 (Mild) to 10 (Very persistent)”), awareness of tinnitus (“Assuming that the whole day (without sleep) is 100%, please state on average the percentage of the day that you are aware of your tinnitus”), and time since onset of tinnitus.

#### Hearing profile

**Figure 1:**
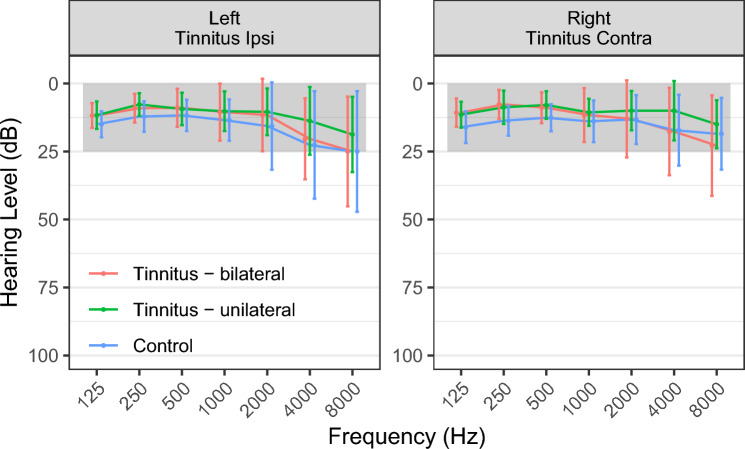
Average audiograms for each group with standard deviations marked as vertical error bars. The gray rectangle represents normal hearing according to WHO^63^; left = left ear, right = right ear, tinnitus ipsi—the ear corresponding to the side of tinnitus in the TU group; for 4 TU participants that were experiencing tinnitus on the right side, right ear hearing levels were combined with left-ear hearing levels of other participants as *tinnitus ipsi*.

The three included groups of participants (two tinnitus subgroups and healthy controls) were not different in terms of their hearing loss characteristics. Mean group hearing loss, for each frequency range from 125 to 8000 Hz for all groups was within the normal range according to WHO^63^. Figure 1 shows mean group audiograms. Several individuals in each group had some degree of hearing loss, see individual PTA and HTA levels for each participant in Supplementary Figs. 1 and 2. Two participants from the healthy control group did not undergo the tonal audiometry test due to time constraints but had no history of hearing problems. None of the participants was using any audiological devices (i.e. e.g. cochlear implants or hearing aids) prior or during the study. All participants used spoken communication and had no diagnosed problems with language development or speech understanding.

In order to better characterize the hearing profile of the participants, PTA and HTA values were compared across groups separately for the right ear (or contralateral to tinnitus), the left ear (or ipsilateral to tinnitus), the better ear, the worse ear, and the hearing level asymmetry (difference between the ears). The results are presented in Supplementary Table 1 and show no significant between-group differences.

#### General psychological questionnaires

All participants completed two screening questionnaires (except for one person from the control group and one person from the TU group) referring to their psychological functioning.

1. *State-Trait Anxiety Inventory (STAI)* measuring anxiety tendencies which includes 40 questions, 20 referring to anxiety as a state and 20 referring to anxiety as a trait. The respondent answers the questions with respect to the extent that they refer to him/her behavior and personality. The minimum row points that can be obtained in each scale is 20 and the maximum is 80. The scores are summed up and converted to a sten scale. According to the generally accepted norms, a score corresponding to 1st-3rd sten is considered *very low* or *low*, a score at the level of 4th-6th sten is considered *average*, and a score at the level of 7th-10th sten is considered *elevated, high* or *very high*^64,65^.
2. *Depression Assessment Questionnaire* (DAQ, the original Polish name: “Kwestionariusz do Pomiaru Depresji”) measuring depression tendencies using 75 statements. The respondent chooses one of 4 answers: (1) never, (2) sometimes, (3) often, (4) always or (1) very much, (2) significantly, (3) slightly, (4) not at all, with respect to how much a given statement refers to him/herself. The maximum overall score is 240 (the minimum is 0). The scores are summed up and converted to a sten scale. According to generally accepted norms, a score corresponding to 1st-3rd sten is considered *very low* or *low*, a score at the level of 4th-6th sten is considered *average*, and a score at the level of 7th-10th sten is considered *elevated, high* or *very high*^66^.

#### Ethics statement

All study participants signed an informed consent to take part in the study and were not compensated for participation. The study was approved by the Bioethical Committee of the Institute of Physiology and Pathology of Hearing (number of approval KB.IFPS.13/2016 with changes KB.IFPS.29/2017) and conformed to the Declaration of Helsinki from 2013.

### MRI and MRS data collection

MRS data for the experiment had been collected in the years 2018–2020. All participants were scanned in a 3T Siemens Prisma FIT scanner (Siemens Medical Systems, Erlangen, Germany) at the Bioimaging Research Center of the Institute of Physiology and Pathology of Hearing, with a 20-channel receiver head-coil. A single voxel spectroscopy (SVS) PRESS (Point-Resolved Spectroscopy Sequence) sequence was applied for collection of MRS data, using standard Siemens water suppression (Water Saturation, 50 Hz BW) and no lipid suppression. MRS data was collected from four cubic 3.75 cm^3^ (1.5 cm × 1.5 cm × 1.5 cm) regions-of-interest in the brain, placed in the left temporal lobe, right temporal lobe, left frontal lobe, right frontal lobe. The MRS sequence parameters were: TR (time of repetition) = 2000 ms, TE (time of echo) = 40 ms, TA (time of acquisition) = 4 min 26 s, 128 averages with 1024 time points and 1200 Hz bandwidth. For single voxel localization, an anatomical T2-weighted scan was performed in three orthogonal sequences: coronal (TR = 5000 ms, TE = 98 ms, slice thickness 4 mm, FOV 197 × 220), transverse (TR = 4000 ms, TE = 88 ms, slice thickness 4 mm, FOV 197 × 220) and sagittal (TR = 3000 ms, TE = 111 ms, slice thickness 4 mm, FOV 220 × 220), for the total scanning time of 8 min.

**Figure 2:**
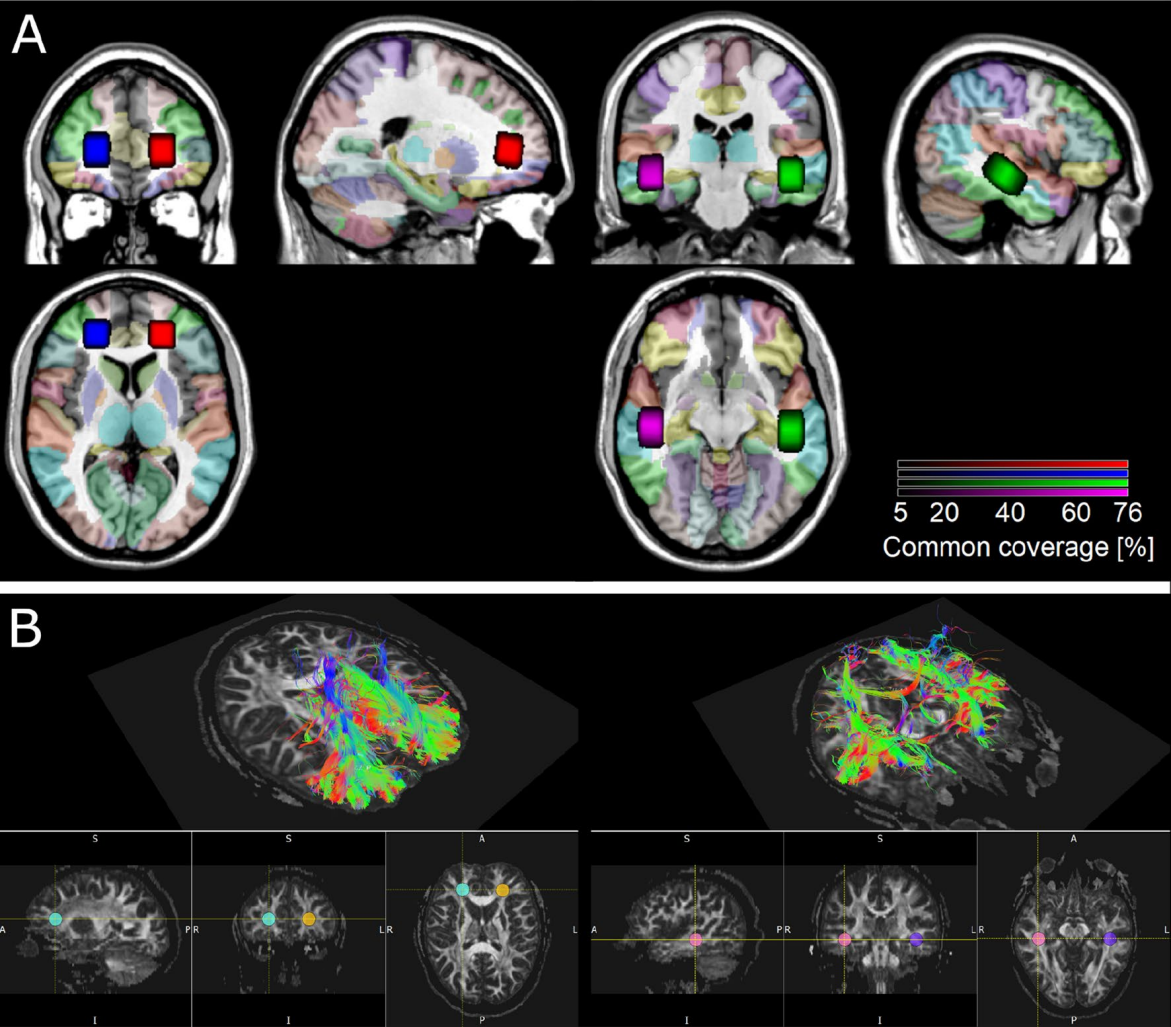
(**A**) Mean group locations of the 4 selected regions of interest (ROIs): left frontal (red), right frontal (blue), left temporal (green), right temporal (purple); shown in MNI space over Automated Anatomical Labeling atlas (AAL) parcellation. Voxel brightness encodes the overlap of the voxel locations across subjects. Semi-transparent colors represent the atlas parcellation. Image prepared in MRIcroGL, using Colin 27 brain as a template^67,68^. Based on AAL, temporal ROIs included bilateral posterior superior and medial temporal lobes (including higher-order auditory cortex), and the frontal ROIs containing parts of bilateral frontal poles (files with mean group ROI masks are included in *Supplementary Materials*). (**B**) Reconstruction of white matter tracts in 4 ROIs in an example single-subject brain, using FACT – Fiber Assignment by Continuous Tracking^69^. A Julich DTI atlas^70–72^ was applied to identify white matter tracts in each ROI. Frontal ROIs contained parts of forceps minor (FM), anterior corona radiata (ACR), cingulum and corpus callosum (CC). Temporal ROIs uncinate fascicle (UF), fronto-occipital fascicle (FOF), inferior longitudinal fascicle (ILF), middle longitudinal fascicle (MLF) (https://fsl.fmrib.ox.ac.uk/fsl/fslwiki/Atlases).

An experienced MRI technician located 4 voxels corresponding to the 4 regions of interest (ROIs) manually for every person, using the coronal, the sagittal and the transverse T2 plane images, in temporal and frontal lobes. The locations were chosen to minimize the content of the cerebrospinal fluid, blood vessels, bones and air cavities. The size of the voxel (3.75 cm^3^) used in the study was optimized to fit various individual brain anatomies (i.e. head sizes and relative distances from brain structures) and at the same time to maintain a reasonable scan duration. Shimming of the MRS data was automatic. Figure 2 depicts group-level brain coverage of the selected voxels and a model of white matter tracts crossing through the selected regions of interest.

## Data analysis

### MRS spectra quality and statistical analysis

#### Selection of metabolites

Raw data from each voxel in each participant was analyzed with LCModel^73^ version 6.3-1R. The analysis of spectra focused on the following components: Glx, tNAA (total NAA), tCho (total Cho), mI (myoinositol). Glx is a composite measure of glutamate (Glu) and glutamine (Gln), metabolites with a significant spectral overlap^74^. Similarly, tNAA is a composite measure of N-acetylaspartate (NAA) and N-acetylaspartylglutamate (NAAG), and tCho is composed of phosphocholine (PCh) and glycerophosphocholine (GPC)^29^. Metabolite levels were further analyzed as ratios to tCr (total creatine) levels, e.g. Glx/tCr.

#### Spectra quality control

The MR technician made sure during data collection that signal-to-noise ratios (SNR) for all of the spectra exceeded the value of 3 and only spectra with a linewidth (FWHM) below 0.1 ppm remained, according to the general MRS spectra quality recommendations^75^. Following further guidelines for the LCModel, spectra with Cramér-Rao lower bounds of standard deviation (CRLB) above 15% were removed. Representative spectra for each of the three study groups have been presented in Supplementary Materials, Fig. S3.

#### Tissue segmentation

As part of further quality control, tissue segmentation was performed using high-resolution 3D T1 images of the study subjects, to white matter (WM), gray matter (GM) and the cerebrospinal fluid (CSF). Two participants did not undergo this additional T1 exam and so the tissue segmentation was not performed. The individual T1 and T2 images (the latter collected during the MRS session) were coregistered using affine co-registration. Voxel dimensions from the SVS MRS files metadata were used as binary masks on the T1 images which were segmented in SPM12 (SPM12 Software—Statistical Parametric Mapping; www.ucl.ac.uk) to obtain probabilistic maps of WM, GM and the CSF content, for each person and voxel individually, for 4 locations (left and right temporal lobe, left and right frontal lobe). The mean group content of the tissue types was compared between the three groups using a Kruskal–Wallis test.

#### Statistical analysis of the MRS spectra

The levels of each metabolite were summarized. One-way Welch’s ANOVA was applied to assess differences between concentrations in the three groups of participants (with no tinnitus, unilateral tinnitus, bilateral tinnitus) in each of the 4 brain regions separately. This was followed by planned post-hoc t-tests to compare the measured metabolite levels between the groups. Normality was inspected for each group’s results distribution, using the Shapiro–Wilk test.

### Statistical analysis of the questionnaire scores

The scores of the THI, TFI, DQ, STAI questionnaires and scales of awareness, persistence and duration of tinnitus were summarized for each group separately and the scores were compared between the groups using a Kruskal–Wallis test, followed by Wilcoxon rank sum tests (if the omnibus test was significant). Severity levels based on THI and TFI scores were also analyzed using Fisher’s exact test.

### Correlation analysis between PTA/HTA values, Glx/tCr levels, and Chol/tCr levels

Concentrations of Glu^33,76^ and Cho^19^ in regions of interest including the auditory cortex have been shown to correlate with hearing loss levels (and thus potentially confounding the effect of tinnitus per se). We verified whether this effect exists in our data by testing for Spearman correlations between Glx and Cho levels and PTA/HTA values derived from an audiometric evaluation.

### Tools for analysis

For all the described analyses we used the following software: base R^77^ and tidyverse^78^ packages for statistical analyses, FID-A for file conversion^79^, gtsummary^80^ to create tables, ggbeeswarm^81^ and ggsignif^82^ for visualization.

## Results

### Psychological and tinnitus questionnaires

**Figure 3:**
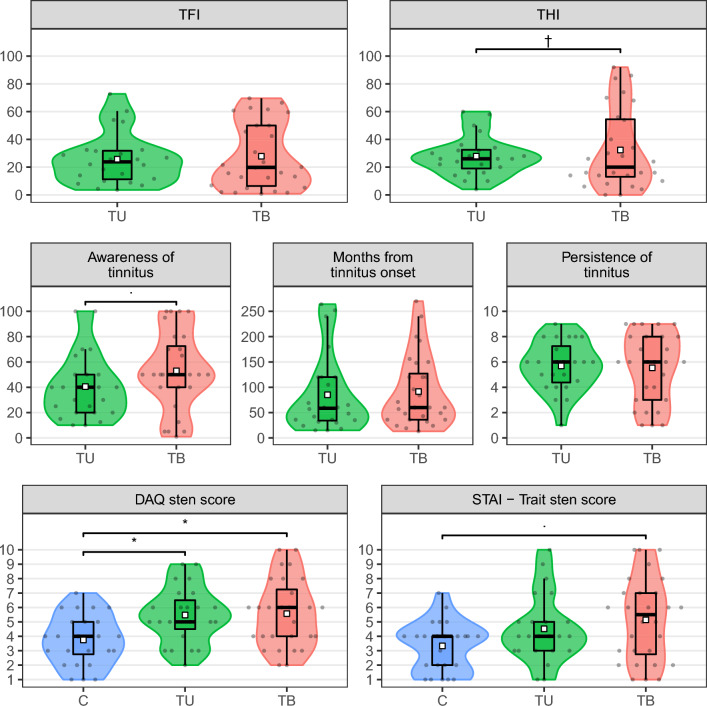
Scores in the tinnitus questionnaires (two upper rows) and the depression/anxiety questionnaires (bottom row). Groups are color coded. Individual subject results are depicted as points in the violin plots. White squares represent group mean values while middle horizontal bars on box plots represent median values. TB—bilateral tinnitus, TU—unilateral tinnitus, C—control, TFI—Tinnitus Functional Index, THI—Tinnitus Handicap Index, DAQ—Depression Assessment Questionnaire, STAI—State-Trait Anxiety Inventory; awareness, duration and persistence were assessed on a VAS scale. For TFI and THI raw scores are represented. DAQ and STAI results are presented on a sten scale. *p < 0.05, *p* < 0.1, †—raw score difference not significant, but categorized results difference significant.

Table 2 and Fig. 3 depict the results of two tinnitus questionnaires applied in the two groups of patients with tinnitus. There were no significant differences found between the raw scores in the two groups in none of the questionnaires (all *p*-values > 0.7, Wilcoxon rank sum test). The TFI mean group scores of 26 and 28 points, in TU and TB, respectively, indicated small or moderate levels of tinnitus severity^60–62^. As for the severity level distribution, in 36% of TB participants tinnitus was significantly severe or worse (above 76 points), as compared to 16% in the TU group, but the difference in problem level occurrence was not statistically significant (*p* = 0.15, Fisher’s exact test). Mean THI scores of 28 and 32, in TU and TB, respectively, indicated mild influence of tinnitus on everyday life. Statistically significantly, 50% of TB participants reported slight influence of tinnitus (0–16 points) and in 18% the influence was mild (18–36 points), whereas in the TU group the proportions were 25% and 58%, respectively (*p* = 0.023, Fisher’s exact test). According to the Polish Tinnitus Characteristics Inventory, in the TB group the mean awareness of tinnitus (during the day) was higher, as compared to the TU group, but the difference was not statistically significant (*p* = 0.09, Wilcoxon rank sum test). There were no effects revealed for any inter-group differences with respect to the duration and persistence of tinnitus.

**Table 2:** Results of tinnitus questionnaires.

Characteristic^a^	TU, N = 24^b^	TB, N = 28^b^	*p*-value^c^
TFI			0.8
Median (IQR)	24 (11–32)	20 (6–50)	
Mean (SD)	26 (18)	28 (24)	
Range	4–73	1–70	
TFI—problem level			0.15
Small	9/24 (38%)	13/28 (46%)	
Moderate	11/24 (46%)	5/28 (18%)	
Significant	3/24 (12%)	8/28 (29%)	
Very significant	1/24 (4.2%)	2/28 (7.1%)	
THI			0.7
Median (IQR)	26 (19–32)	20 (13–54)	
Mean (SD)	28 (14)	32 (29)	
Range	4–60	0–92	
THI—influence level			**0.023**
Slight	6/24 (25%)	14/28 (50%)	
Mild	14/24 (58%)	5/28 (18%)	
Moderate	2/24 (8.3%)	3/28 (11%)	
Severe	2/24 (8.3%)	3/28 (11%)	
Catastrophic	0/24 (0%)	3/28 (11%)	
Persistence of tinnitus			> 0.9
Median (IQR)	6.00 (4.38–7.25)	6.00 (3.00–8.00)	
Mean (SD)	5.69 (1.95)	5.54 (2.77)	
Range	1.00–9.00	1.00–9.00	
Awareness of tinnitus			*0.090*
Median (IQR)	40 (20–50)	50 (40–72)	
Mean (SD)	41 (25)	53 (31)	
Range	10–100	1–100	
Months since tinnitus onset			**0.5**
Median (IQR)	58 (34–120)	60 (36–127)	
Mean (SD)	85 (76)	92 (70)	
Range	15–264	13–270	

^a^SD—standard deviation, IQR—inter-quartile interval.

^b^TU—tinnitus unilateral, TB—tinnitus bilateral; for TFI and THI levels: n/N (%); n—subgroup count, N—whole group count; Fisher’s exact test.

^c^For continuous variables: Wilcoxon rank sum test; For categorical variables: Fisher’s exact test.

*p* < 0.1 are in italics, *p* < 0.05 are in bold.

Table 3 and Fig. 3 depict the results of the DAQ and STAI questionnaires in three study groups. The mean results for both DAQ and STAI-trait questionnaires were in the range of 4–6 sten in all three groups which indicated average levels of the reported depressive symptoms and anxiety, respectively. Nevertheless, both TU and TB groups had statistically significantly higher DAQ scores than the healthy control participants (*p* = 0.01 and *p* = 0.023 respectively, Wilcoxon rank sum test). Also in the STAI-trait questionnaire, the scores were higher in the TB group, as compared to the control group, at a tendency level (*p* = 0.063, Wilcoxon rank sum test). Both for DAQ and STAI, only in the control group there was no participant with scores beyond the 7th sten, indicating elevated/high levels of symptoms.

**Table 3:** Results of psychological questionnaires.

Characteristic^a^	C, N = 25^b^	TU, N = 24^b^	TB, N = 28^b^	*p*-value^c^	Post-hoc tests		
					C versus TU^d^	C versus TB^d^	TU versus TB^d^
DAQ sten score				**0.007**	**0.017**	**0.023**	> 0.9
Median (IQR)	4.00 (2.75–5.00)	5.00 (4.50–6.50)	6.00 (4.00–7.25)				
Mean (SD)	3.75 (1.75)	5.48 (1.95)	5.57 (2.36)				
Range	1.00–7.00	2.00–9.00	2.00–10.00				
Missing	1	1	0				
STAI—Trait sten score				*0.060*	0.4	*0.063*	> 0.9
Median (IQR)	4.00 (2.00–4.00)	4.00 (3.00–5.00)	5.50 (2.75–7.00)				
Mean (SD)	3.33 (1.71)	4.52 (2.33)	5.14 (2.88)				
Range	1.00–7.00	1.00–10.00	1.00–10.00				
Missing	1	1	0				

^a^SD—standard deviation, IQR—inter-quartile interval.

^b^C—control, TU—tinnitus unilateral, TB—tinnitus bilateral.

^c^Kruskal–Wallis rank sum test.

^d^Wilcoxon rank sum test; Bonferroni correction.

*p* < 0.1 are in italics, *p* < 0.05 are in bold.

### MRS spectra after quality control

For Glx, 294 observations remained ultimately, including 77 from the left frontal voxel, 76 from the right frontal voxel, 70 from the left temporal voxel and 71 from the right temporal voxel. A summary of all the quality control values for Glx and tCr is presented in Supplementary Tables 2 and 3. The CRLB values and the number of rejected observations for metabolites assessed as part of an exploratory analysis (mI, tNAA, tCho) are presented in Supplementary Table 5.

### Results of tissue segmentation

Tissue segmentation showed that all 4 voxels/regions of interest mainly consisted of white matter (61–97% in frontal regions, 43–80% in temporal regions; see details in Supplementary Table 4) and there were no differences between the three groups with respect to the percentage content of different types of tissue (all *p*-values > 0.4, Kruskal–Wallis rank sum test).

### MRS results

**Figure 4:**
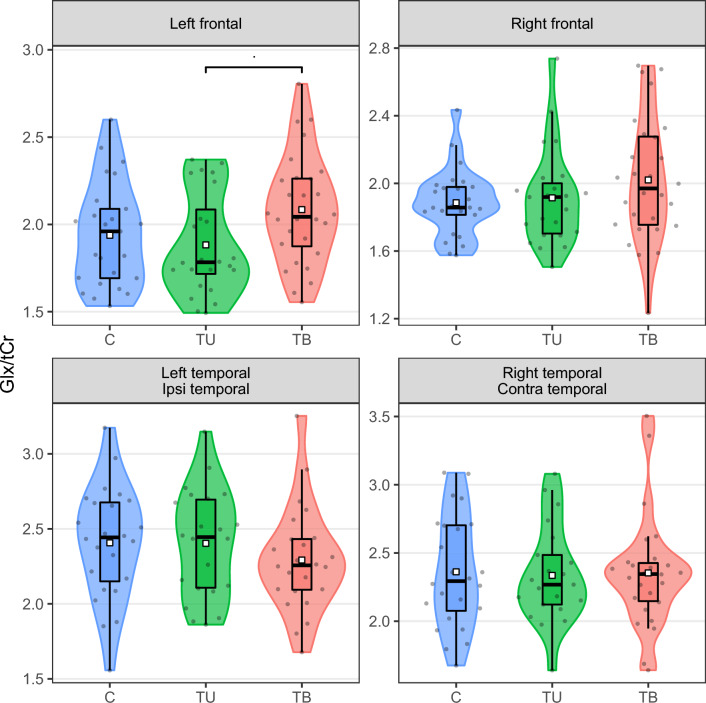
Glx/tCr levels in the four ROIs. Individual subjects are depicted as points in the violin plots. White squares represent group mean values while middle horizontal bars on box plots represent median values. TB—bilateral tinnitus, TU—unilateral tinnitus, C—control; Ipsi—the side corresponding to the side of tinnitus in the TU group (for 4 TU participants that were experiencing tinnitus on the right side, the sides were switched), Contra—side opposite to tinnitus laterality. *p* < 0.1.

Table 4 and Fig. 4 show levels of Glx/tCr in three groups separately. ANOVA revealed a tendency for a difference in levels in the left frontal ROI (*p* = 0.055) between three study groups. Results of planned post-hoc tests showed a tendency for increased Glx/tCr concentration in the TB group, as compared to the TU group in the left frontal ROI (*p* = 0.058). As depicted, no statistically significant differences were found between the groups in the temporal ROIs and the right frontal ROI (all *p*-values > 0.058, Welch t-test, Bonferrroni correction). There was also no between-group difference in inter-hemispheric differences of Glx levels in temporal ROIs (*p* = 0.8, Welch ANOVA). The results for tNAA, tCho, mI showed no statistically significant differences between the groups either and have been presented in Supplementary Fig. 4 and Supplementary Tables 6–8.

**Table 4:** Glx/tCr levels in four regions of interest compared across groups.

Region^a^	Descriptive statistics			Welch ANOVA				Post-hoc tests		
	C, N = 25^b^	TU, N = 24^b^	TB, N = 28^b^	F	p	df1	df2	C versus TU^c^	C versus TB^c^	TU versus TB^c^
Left frontal				3.1	*0.055*	2.00	49.1	> 0.9	0.3	*0.058*
Median (IQR)	1.96 (1.69–2.09)	1.78 (1.72–2.09)	2.04 (1.87–2.26)							
Mean (SD)	1.94 (0.30)	1.88 (0.29)	2.08 (0.31)							
Range	1.53–2.60	1.49–2.37	1.56–2.81							
S–W test	0.16	0.01	0.77							
Missing	0	0	0							
Right frontal				1.4	0.3	2.00	45.9	> 0.9	0.3	0.8
Median (IQR)	1.86 (1.81–1.98)	1.92 (1.70–2.00)	1.97 (1.76–2.28)							
Mean (SD)	1.89 (0.20)	1.91 (0.29)	2.02 (0.37)							
Range	1.58–2.43	1.51–2.74	1.23–2.70							
S–W test	0.18	0.04	0.43							
Missing	0	1	0							
Left/Ipsi temporal				0.78	**0.5**	2.00	44.5	> 0.9	0.8	0.9
Median (IQR)	2.44 (2.15–2.68)	2.45 (2.11–2.70)	2.26 (2.09–2.43)							
Mean (SD)	2.41 (0.38)	2.40 (0.36)	2.29 (0.35)							
Range	1.56–3.17	1.86–3.15	1.68–3.25							
S–W test	0.98	0.46	0.42							
Missing	1	2	4							
Right/contra temporal				0.03	> 0.9	2.00	45.1	> 0.9	> 0.9	> 0.9
Median (IQR)	2.29 (2.08–2.70)	2.27 (2.12–2.49)	2.35 (2.15–2.43)							
Mean (SD)	2.36 (0.41)	2.34 (0.35)	2.35 (0.42)							
Range	1.68–3.09	1.64–3.08	1.64–3.50							
S–W test	0.43	0.47	0.01							
Missing	1	3	2							

^a^SD—standard deviation, IQR—inter-quartile interval, S–W test—Shapiro–Wilk test of normality result (*p*-value), Ipsi—side ipsilateral to the perceived unilateral tinnitus, Contra—side contralateral to the perceived unilateral tinnitus.

^b^C—control, TU—tinnitus unilateral, TB—tinnitus bilateral.

^c^Welch t test; Bonferroni correction.

*p* < 0.1 are in italics, *p* < 0.05 are in bold.

### Correlation analysis between hearing levels (HTA and PTA) and metabolite levels (Glx/tCr tCho/tCr)

There was no statistically significant correlation revealed between the hearing levels (PTA and HTA) of the participants and the levels of Glx/tCr and tCho/tCr (as measured by Spearman’s ρ correlation test) in temporal ROIs. There was a significant correlation found between left/ipsilateral ear HTA levels and tCho/tCr in frontal ROIs. The results are presented in Supplementary Figs. 5–8.

## Discussion

### Motivation and main results

Magnetic resonance spectroscopy (MRS) remains a valuable non-invasive technique to study tinnitus, a condition whose exact pathomechanisms and the underlying neuronal correlates are still not fully understood. Our main aim was to expand the existing limited knowledge regarding involvement of glutamate/glutamine (basic excitatory neurotransmitter in CNS) and other metabolites’ concentration changes in the occurrence of subjective tinnitus. To the best of our knowledge, our work is the second one, following Isler and colleagues^33^, which measured glutamate concentration with MRS in people with tinnitus and a control group. We are also the first ones to investigate metabolite levels in the temporal brain areas excluding primary auditory cortex (complementary to other existing works) in a sizable cohort of 52 participants, divided into unilateral vs bilateral tinnitus subgroups. Our results failed to demonstrate any significant relationship between tinnitus and Glx/tCr concentration in the temporal brain region of interest which excluded the primary auditory cortex. Tinnitus was also not linked to the concentration of any other neurometabolites, which may indicate a generally good CNS health of our participants. Unexpectedly, however, we found a tendency towards higher Glx/tCr levels in participants with bilateral tinnitus, as compared to those with unilateral tinnitus, in the reference left prefrontal brain area.

### No significant effect of tinnitus in the temporal lobe excluding the primary auditory cortex

In our study the selected region of interest in bilateral temporal lobes encompassed parts of the high-order auditory cortex and the related white matter tracts, shown to be engaged in conscious auditory perception (such as in tinnitus, but also during auditory hallucinations and auditory imagery), as well as in auditory memory^6,40–42^^.^. With respect to tinnitus specifically, superior/medial temporal lobes have also been indicated as engaged in resolving the conflict between auditory memory of sounds and the distorted signal derived from the damaged peripheral auditory system, thereby leading to the tinnitus percept^6^. Beyond that, the included white matter tracts, such as the uncinate fascicle (UF), the fronto-occipital fascicle (FOF), and the inferior and middle longitudinal fascicles (ILF, MLF), as well as the gray matter functional regions of the superior and medial temporal lobes, have been shown to be involved in episodic memory, language content processing, and learning; in MLF and FOF through their connections with the Heschl gyrus and in UF and FOF through their connections to the prefrontal lobe^83–87^.

On the basis of given literature, we expected that some differences would occur in the chosen regions of interest. Nevertheless, the presence of tinnitus and its laterality were not associated with different levels of Glx in these parts of the temporal lobes. Our results may therefore suggest that physiological and metabolic/neurotransmitter changes accompanying tinnitus are only present or are more profound in the primary auditory cortex (including the Heschl gyrus), as indicated in previous animal and human research^16,19–25,30,32,33,88,89^.

In our study we have not found a significant relationship between hearing levels (both in low-middle and high frequencies; and with all participant groups matched in that respect) and metabolite concentrations levels in the selected temporal regions. Other works in tinnitus investigating regions of interest including the primary auditory cortex found such correlations (e.g. Isler et al.^33^, and Yoo et al.^90^). Therefore, we can assume that the hearing status did not meaningfully affect the lack of between-group differences in the analyzed metabolite levels in the temporal lobe outside the primary auditory cortex.

As a future direction, adding another region of interest in the primary auditory cortex, could complement the current study design, to investigate the possible dissociation between the primary and higher-level auditory regions, and the potential confounding effect of hearing loss on metabolite levels.

### Prefrontal brain regions in tinnitus: attention, stress and psychological well-being

Unexpectedly, we have found an effect in Glx levels in one of the reference prefrontal brain regions. Nevertheless, this finding is in line with the growing number of studies providing evidence that tinnitus maintenance and its perceptual aspects involve neuronal networks beyond the auditory system and the primary auditory cortex^50,91^.

Frontal brain areas have been indicated in tinnitus pathophysiology, including in white matter tracts^44,90,92–94^. Frontal pole in particular (constituting approximately 15% of both frontal regions of interest) has been suggested as a neuronal correlate of tinnitus awareness and annoyance^95^, as well as the tinnitus perception network^96^. Furthermore, distorted communication between the prefrontal and auditory cortex through the fibers crossing the frontal ROI (CC, cingulum and ACR) has been suggested as contributing to chronic tinnitus^6,19,97^ (see e.g. work by Chen and colleagues^93^ who describe similar effects with respect to functional connectivity in tinnitus, as measured with resting state functional magnetic resonance imaging).

With respect to the specific patomechanisms, one line of research postulates that subsequent to a peripheral auditory damage, the maintained awareness of tinnitus is due to abnormal engagement of cognitive control and impaired attention-switching mediated by prefrontal cortex (and the limbic system)^47,91,94,98–100^. According to another yet related hypothesis, the prefrontal cortex is part of a noise canceling/inhibition system (which also includes the thalamic reticular nucleus adjacent to the auditory thalamus, MGB) whose permanent failure leads to tinnitus becoming chronic^6,47,91,101,102^.

Specifically in our study, Glx levels were higher in bilateral as compared to unilateral tinnitus in the *left* prefrontal region. As proposed by Palmiero and colleagues^103^, this region has been shown to be engaged in executive control and inhibition of negative (vs positive) distractors. We can thus speculate that the relationship between Glx levels and tinnitus laterality is due to these functions being more efficient in unilateral tinnitus, which might have also prevented development of a bilateral tinnitus percept (see an indication for impaired selective attention in bilateral tinnitus in Sharma et al.^104^, and Bartnik et al.^105^). In other words, the perceived laterality of tinnitus may be mediated by the frontal lobe.

At the same time, there were no differences shown in hearing level asymmetry (neither in low-middle frequency ranges nor in high frequencies) between the two tinnitus groups that might otherwise potentially account for the perceived tinnitus laterality (cf. Genitsaridi et al.^34^).

In the current study, all participants with diagnosed affective diseases have been excluded. Nevertheless, both people with unilateral and bilateral tinnitus showed slightly higher average levels of depressive symptoms than the healthy controls, with about 25% individuals in both tinnitus groups presenting high symptom levels. With respect to the anxiety screening tool, participants with tinnitus perceived on both sides had higher levels of the symptoms than the control group (at a tendency level). Furthermore, although these effects were not statistically significant, in the bilateral vs unilateral group there were proportionally more participants assessing the perceived tinnitus-related handicap as high, and this group was also more aware of their tinnitus in their daily life. In the existing literature, most studies included participants with the bilateral type of tinnitus (which is more prevalent) or participants with mixed lateralities. Few existing epidemiological studies comparing the two types of tinnitus, converging with our results, indicated more annoyance^34,104^, tinnitus handicap^106^ (although see work by Song et al.^36^ who found a reverse effect) and depressive symptoms^106^ when tinnitus was perceived bilaterally^52^.

The small differences between the two subgroups in our study could potentially contribute to the metabolite level differences observed in the left frontal voxel. In fact, a relationship between psychological functioning and glutamate levels in frontal brain regions has been suggested in people with depressive disorders, including the prefrontal cortex, as well as in the integrity of CC and ACR white matter tracts, both included in our frontal regions of interest (see e.g. a review by Luttenbacher and colleagues^107^). Changes in glutamate levels in the medial prefrontal cortex have also been suggested to reflect chronic stress exposure and stress adaptation^92,108–110^.

On the basis of our results and literature, future MRS studies could focus more specifically on frontal brain regions, including the dorso-lateral prefrontal cortex (DLPFC) and ventro-medial-prefrontal cortex (vmPFC), both indicated in emotional processing and cognitive control in tinnitus^111,112^.

### Tinnitus heterogeneity and participants’ inclusion criteria

Tinnitus poses a significant research challenge as it is an extremely heterogeneous condition, both with respect to its origin, possible manifestations and comorbidity with hearing loss. Despite the high prevalence of tinnitus worldwide (an estimated 10–25% of the population worldwide), there is no one-size-fits-all treatment available at the moment^27,105,113–116^.

In our study, with the aim to examine the relationship between glutamate (and other metabolites) levels and tinnitus per se, we applied several exclusion criteria including comorbid medical conditions, as well as the diagnosed psychological and psychiatric disorders. To avoid further confounding effects, the three study groups were also similar with respect to age and PTA/HTA values (although we failed to exclude hearing loss cases completely), as both these factors have been found to influence metabolite levels in CNS^76,117–120^. We were also the first ones in human MRS research, to divide the groups based on tinnitus status and laterality. In the future, further subtypes of tinnitus could be distinguished (e.g. based on the perceived type of sound, loudness, distress levels, duration of tinnitus, awareness, participation in tinnitus therapy/training, depression and anxiety levels, etc.). Specifically in bilateral tinnitus, we have found that both the scores in the psychological questionnaires and the relative metabolite levels were consistently more sparsely distributed, as compared to the unilateral tinnitus group and the healthy subjects, which might have been to some other non-defined characteristics of the participants. In order to further elucidate the role of hearing loss in tinnitus generation/maintenance, a detailed broad-band tonal audiometry would allow more precise hearing level measurements in future MRS studies^49,121,122^.

### MRS data quality control

Changes in brain metabolite levels, as measured with MRS, might be very subtle and prone to numerous confounding effects intrinsic to MR imaging, e.g. signal originating from tissues of no interest, subject’s movement or magnetic field inhomogeneity^123^. In our study, before applying any statistical analyses, we therefore performed a data quality check (see *Methods* regarding SNR and FWHM of the acquired metabolite spectra, CRLB of the estimated metabolite levels, WM/GM tissue composition). We also used a Siemens PRESS sequence with the TE value optimized to measure Glx concentration^74^. In addition, the MRS data was collected from relatively small localized ROIs. The voxel size was small in order to achieve a high-quality signal, by placing it far from the skull and the sinuses (a considerable challenge in MRS studies of the prefrontal cortex and the temporal lobes^75^), while also taking into account different head sizes and anatomy. The resulting ROIs contained a considerable amount of white matter (WM) tissue^37,39^, where, as recent data shows, glutamate supports myelination and glial receptor function which then enhances axon potential initiation and propagation of impulses^124–128^. Since WM and GM tissue have been shown to affect the MRS signal differently^37–39^, it was confirmed that the groups in the study were not different in that aspect. We were unable to report levels of GABA in the same regions of interest as for Glx and other metabolites, although it might have provided valuable information regarding the excitation-inhibition mechanisms in tinnitus. The reason was, nevertheless, lack of a dedicated commercial MRS sequence for the 3T Siemens PRISMA scanner at the time of the study (such as e.g. MEGA PRESS). This limitation will be addressed in our future studies.

### Challenges of the MRS technique and future developments

There is considerable diversity in the applied MRS methods in today’s research, both in terms of the scanning sequences as well as the data analysis. As an example, in each of the other three published MRS studies in tinnitus, the authors applied a different MRS sequence available for 3T MRI scanners (STEAM, MEGA-PRESS, 2D-JPRESS), used different software for data analysis (LCModel, Gannet, ProFit) and applied various criteria for data quality check. It has been shown that all these aspects significantly contribute to the MRS measurements^127,129^. Future studies might benefit from higher replicability, with uniform MRS research protocols, including optimal scanning sequences and quality measures. There are several expert consensuses and recommendations for advanced MRS applications issued only recently^130–132^. Beyond the mentioned issues, discrepancies exist considering the size of the region of interest for single-voxel spectroscopy. Some authors opt for using large voxels for local metabolite measurements (e.g. in tinnitus MRS studies: Isler et al.^33^; Sedley et al.^19^) that, however, do not warrant high regional specificity and increase the risk of contaminating the MRS data with signals from tissues of no interest (e.g. cerebro-spinal fluid, skull, air). Others use voxels of smaller sizes, which approach nevertheless requires signal-to-noise ratio to be improved, e.g. by repeated measurements (e.g. our study and Cacace et al.^32^) as well using MR scanners with high field homogeneity. In addition, the reliance on a single voxel (as in SVS MRS) yields its placement a crucial part of the design and becomes a source of variability in the published results^133^. The emerging whole-brain MRS (MRS imaging, MRSI), with the data collected from multiple small regions of interest simultaneously, can become an alternative to SVS MRS in that respect, although the availability of vendor-provided advanced sequences for MRSI is limited and the technology requires considerably more expertise in data quality assessment and interpretation^131,134^. Finally, recent advances in MRS sequence development and implementation in several commercial devices^135^, new signal processing methods^136^, real-time movement correction algorithms^137^ and application of high-field 7T MR scanners might all improve the reliability and reproducibility of MRS measurements in the future.

## Conclusion

This is the first MRS study investigating brain metabolite levels outside the primary auditory cortex in the context of tinnitus and no severe hearing loss. There was no relationship found between unilateral and bilateral tinnitus, and Glx, tNAA, tCho and mI levels in the temporal areas. Nevertheless, the results suggest that glutamate concentration in the left frontal lobe may play a role in the development and maintenance of bilateral *vs* unilateral subjective tinnitus. The potential mechanisms can include the functioning of the frontal-limbic-auditory noise canceling/inhibition system, attention control, as well as psychological aspects of tinnitus. Further research is necessary to explore the relationship between the tinnitus percept and the neurotransmitter systems in other brain locations. Emerging methodological advances in MRS may facilitate the process.

### Supplementary Information

Supplementary Information.

## Data Availability

Anonymized raw MRS spectra have been made available at Zenodo (doi: 10.5281/zenodo.8379543).
