# Supplementary material for: An MR spectroscopy study of temporal areas excluding primary auditory cortex and frontal regions in subjective bilateral and unilateral tinnitus
Source: Sci Rep. 2023 Oct 27;13:18417. doi: 10.1038/s41598-023-45024-3 (PMC10611771; doi:10.1038/s41598-023-45024-3)
Supplement: Supplementary file 1 — Supplementary Information. [file 41598_2023_45024_MOESM1_ESM.docx]

**Supplementary material: An MR spectroscopy study of temporal areas excluding primary auditory cortex and frontal regions in subjective bilateral and unilateral tinnitus**

Joanna Wójcik^1,*^, Bartosz Kochański^1,*^,Katarzyna Cieśla^1,*^, Monika Lewandowska^2^, Lucyna Karpiesz^3^, Iwona Niedziałek^3^, Danuta Raj-Koziak^3^, Piotr Henryk Skarżyński^4,5,6^, Tomasz Wolak^1^

* equal authorship

Corresponding author: dr Katarzyna Cieśla ([kasia.j.ciesla@gmail.com](mailto:kasia.j.ciesla@gmail.com))

1. Bioimaging Research Center, World Hearing Center, Institute of Physiology and Pathology of Hearing, Mokra 17 Street, Kajetany 05-830 Nadarzyn, Poland;
2. Institute of Psychology, Faculty of Philosophy and Social Sciences, Nicolaus Copernicus University, Fosa Staromiejska 1a Street 87-100 Toruń, Poland;
3. Tinnitus Department, World Hearing Center, Institute of Physiology and Pathology of Hearing, Mokra 17 Street, Kajetany 05-830 Nadarzyn, Poland;
4. Department of Teleaudiology and Screening, World Hearing Center, Institute of Physiology and Pathology of Hearing, Mokra 17 Street, Kajetany 05-830 Nadarzyn, Poland;
5. Institute of Sensory Organs, Mokra 1 Street, Kajetany 05-830 Nadarzyn, Poland;
6. Heart Failure and Cardiac Rehabilitation Department, Faculty of Medicine, Medical University of Warsaw, Kondratowicza 8 Street, 03-242 Warsaw, Poland

**List of Supplementary Materials:**

**Table S1.** Detailed PTA and HTA values and comparisons between groups.

**Figure S1.** Individual PTA values (500-2000 Hz) for each participant.

**Figure S2.** Individual HTA values (4000-8000 Hz) for each participant.

**Table S2.** Main quality control measurements: Glx/tCr CRLB and whole spectra FWHM and SNR.

**Table S3.** Creatine CRLB values.

**Figure S3.** Representative spectra for each group and studied voxel.

**Table S4.** Brain tissue segmentation (GM and WM percentages) and between-group comparisons.

**Table S5.** ml/tCr, tCho/tCr and tNAA/tCr CRLB values.

**Figure S4.** tNAA/tCr, tCho/tCr and mI/tCr levels in the four ROIs.

**Table S6.** tNAA/tCr levels in four regions of interest compared across the groups.

**Table S7.** tCho/tCr levels in four regions of interest compared across the groups.

**Table S8.** mI/tCr levels in four regions of interest compared across the groups.

**Figure S5.** Scatterplots and Spearman’s ρ tests for correlation between PTA values (500-2000 Hz) in both ears separately and levels of Glx/tCr in each ROI.

**Figure S6.** Scatterplots and Spearman’s ρ tests for correlation between PTA values (500-2000 Hz) in both ears separately and levels of tCho/tCr in four ROIs.

**Figure S7.** Scatterplots and Spearman’s ρ tests for correlation between HTA values (4000-8000 Hz) in both ears separately and levels of Glx/tCr in each ROI.

**Figure S8.** Scatterplots and Spearman’s ρ tests for correlation between HTA values (4000-8000 Hz) in both ears separately and levels of tCho/tCr in four ROIs.

**Supplementary File 1.** (File_1_LF.nii) Group coverage mask for left frontal ROI.

**Supplementary File 2.** (File_2_RF.nii) Group coverage mask for right frontal ROI.

**Supplementary File 3.** (File_3_LT.nii) Group coverage mask for left temporal ROI.

**Supplementary File 4.** (File_4_RT.nii) Group coverage mask for right temporal ROI.

**Table S1.** Detailed PTA and HTA values and comparisons between groups.

| Characteristic^1^ | C, N = 25^2^ | TU, N = 24^2^ | TB, N = 28^2^ | p-value^3^ |
| --- | --- | --- | --- | --- |
| **PTA - right/contra ear** |  |  |  | 0.11 |
| Median (IQR) | 12 (8 - 16) | 10 (6 - 13) | 9 (5 - 12) |  |
| Mean (SD) | 13 (7) | 10 (5) | 11 (9) |  |
| Range | 5 - 35 | 0 - 17 | 2 - 38 |  |
| Missing | 2 | 0 | 0 |  |
| **PTA - left/ipsi ear** |  |  |  | 0.10 |
| Median (IQR) | 12 (8 - 16) | 10 (6 - 12) | 7 (5 - 12) |  |
| Mean (SD) | 14 (9) | 10 (6) | 10 (10) |  |
| Range | 5 - 35 | 0 - 23 | 2 - 43 |  |
| Missing | 2 | 0 | 0 |  |
| **PTA - better ear** |  |  |  | *0.063* |
| Median (IQR) | 10 (7 - 13) | 8 (5 - 10) | 6 (3 - 12) |  |
| Mean (SD) | 12 (7) | 8 (4) | 10 (9) |  |
| Range | 5 - 35 | 0 - 17 | 2 - 38 |  |
| Missing | 2 | 0 | 0 |  |
| **PTA - worse ear** |  |  |  | 0.13 |
| Median (IQR) | 12 (10 - 18) | 12 (8 - 14) | 10 (5 - 12) |  |
| Mean (SD) | 15 (8) | 12 (6) | 12 (10) |  |
| Range | 5 - 35 | 0 - 23 | 2 - 43 |  |
| Missing | 2 | 0 | 0 |  |
| **PTA - right/contra - left/ipsi** |  |  |  | >0.9 |
| Median (IQR) | 0.0 (-1.7 - 1.7) | 0.0 (-1.7 - 3.3) | 1.7 (-0.4 - 3.3) |  |
| Mean (SD) | -0.4 (5.0) | -0.5 (5.9) | 0.8 (2.9) |  |
| Range | -18.3 - 5.0 | -18.3 - 6.7 | -5.0 - 6.7 |  |
| Missing | 2 | 0 | 0 |  |
| **HTA - right/contra ear** |  |  |  | 0.2 |
| Median (IQR) | 15 (11 - 21) | 11 (8 - 15) | 15 (8 - 28) |  |
| Mean (SD) | 18 (12) | 12 (9) | 20 (17) |  |
| Range | 5 - 58 | 0 - 48 | 2 - 58 |  |
| Missing | 2 | 0 | 0 |  |
| **HTA - left/ipsi ear** |  |  |  | 0.3 |
| Median (IQR) | 18 (10 - 29) | 12 (8 - 18) | 15 (10 - 31) |  |
| Mean (SD) | 24 (20) | 16 (12) | 23 (17) |  |
| Range | 2 - 78 | 2 - 50 | 5 - 65 |  |
| Missing | 2 | 0 | 0 |  |
| **HTA - better ear** |  |  |  | 0.3 |
| Median (IQR) | 15 (8 - 21) | 10 (7 - 13) | 14 (5 - 28) |  |
| Mean (SD) | 17 (13) | 12 (10) | 19 (17) |  |
| Range | 2 - 58 | 0 - 48 | 2 - 58 |  |
| Missing | 2 | 0 | 0 |  |
| **HTA - worse ear** |  |  |  | 0.2 |
| Median (IQR) | 18 (12 - 29) | 15 (10 - 18) | 19 (12 - 31) |  |
| Mean (SD) | 25 (19) | 17 (12) | 24 (17) |  |
| Range | 8 - 78 | 5 - 50 | 8 - 65 |  |
| Missing | 2 | 0 | 0 |  |
| **HTA - right/contra - left/ipsi** |  |  |  | 0.6 |
| Median (IQR) | -2 (-9 - 2) | 0 (-6 - 0) | -4 (-6 - 2) |  |
| Mean (SD) | -6 (12) | -4 (9) | -2 (5) |  |
| Range | -48 - 8 | -40 - 5 | -10 - 8 |  |
| Missing | 2 | 0 | 0 |  |
| ^1^SD - standard deviation, IQR - inter-quartile interval, Ipsi – side ipsilateral to the perceived unilateral tinnitus, Contra – side contralateral to the perceived unilateral tinnitus | | | | |
| ^2^C - control, TU - tinnitus unilateral, TB - tinnitus bilateral | | | | |
| ^3^Kruskal-Wallis rank sum test | | | | |


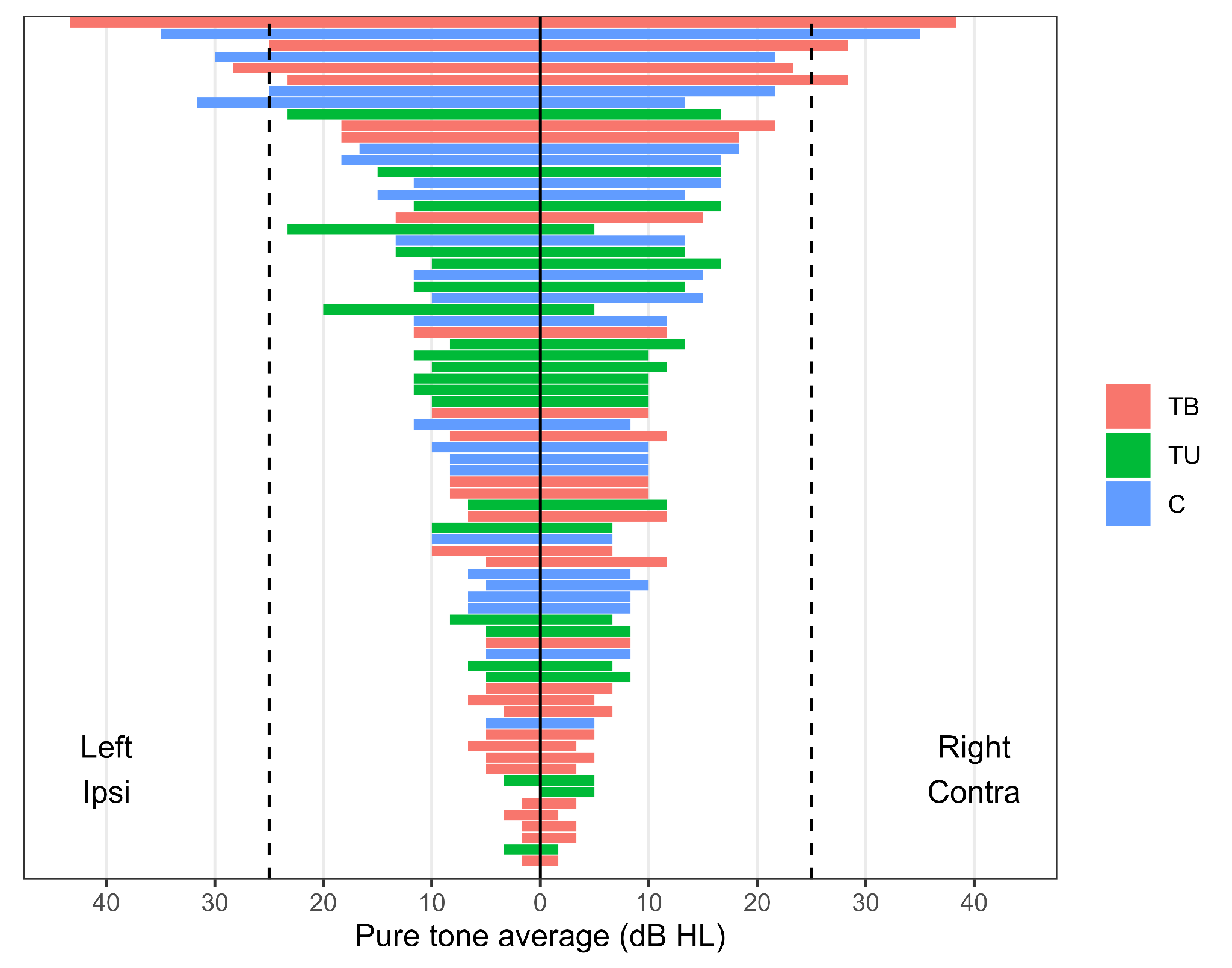


**Figure S1.** Individual PTA values (500-2000 Hz) for each participant. Dashed lines represent the threshold for normal hearing level, Ipsi – side ipsilateral to the perceived unilateral tinnitus, Contra – side contralateral to the perceived unilateral tinnitus.


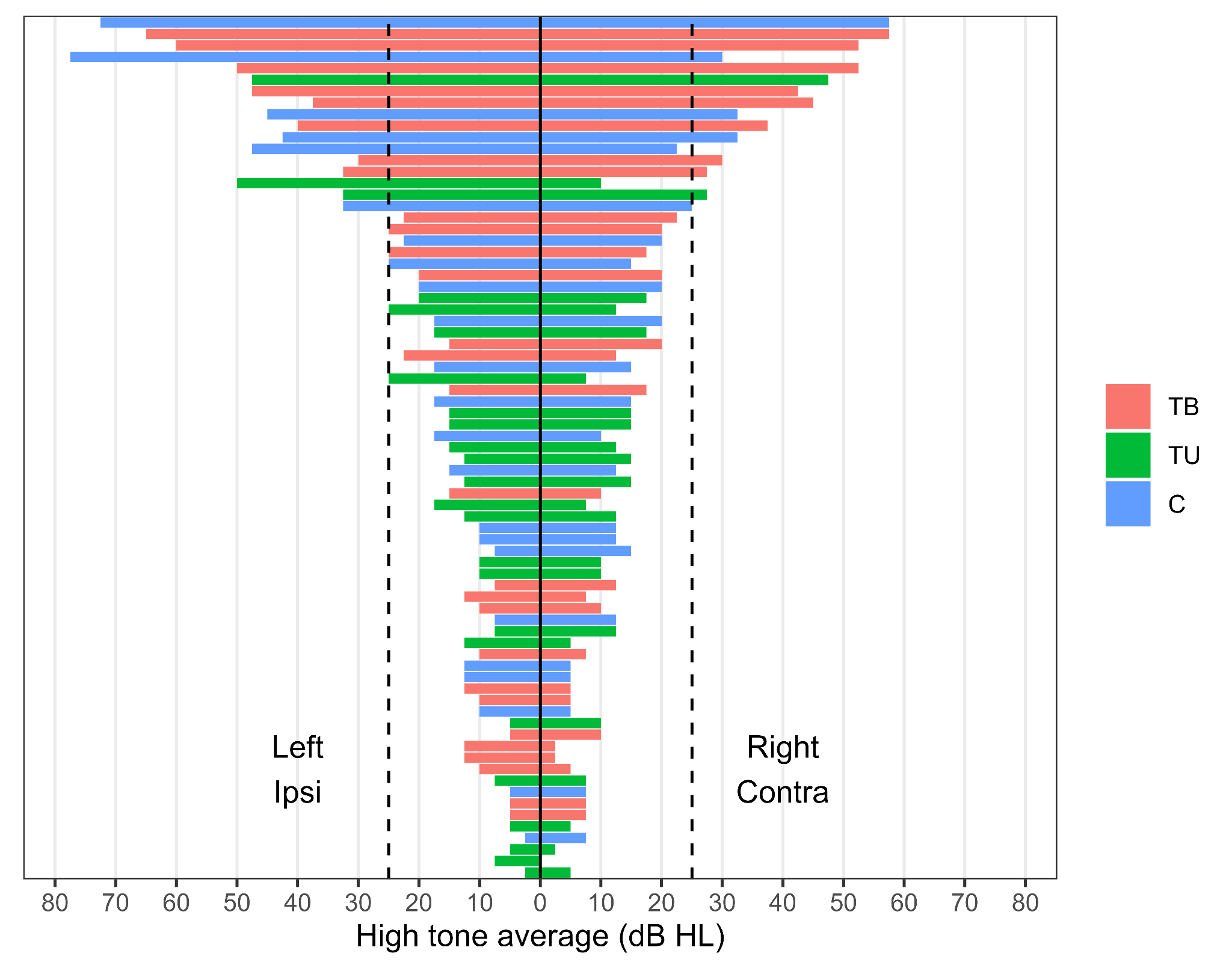


**Figure S2.** Individual HTA values (4000-8000 Hz) for each participant. Dashed lines represent the threshold for normal hearing level, Ipsi – side ipsilateral to the perceived unilateral tinnitus, Contra – side contralateral to the perceived unilateral tinnitus.

**Table S2.** Main quality control measurements: Glx/tCr CRLB and whole spectra FWHM and SNR.

| Region^1^ | Characteristic^1^ | C, N = 25^2^ | TU, N = 24^2^ | TB, N = 28^2^ | p-value^3^ |
| --- | --- | --- | --- | --- | --- |
| Left frontal | **FWHM (ppm)** |  |  |  | 0.9 |
|  | Mean (SD) | 0.050 (0.008) | 0.051 (0.009) | 0.051 (0.009) |  |
|  | Range | 0.038 - 0.067 | 0.038 - 0.067 | 0.038 - 0.076 |  |
|  | **SNR** |  |  |  | 0.2 |
|  | Mean (SD) | 16.08 (1.98) | 15.12 (2.49) | 14.89 (2.70) |  |
|  | Range | 13.00 - 20.00 | 11.00 - 19.00 | 10.00 - 20.00 |  |
|  | **CRLB** |  |  |  | 0.5 |
|  | Mean (SD) | 8.36 (1.08) | 8.83 (1.46) | 8.39 (1.50) |  |
|  | Range | 7.00 - 12.00 | 7.00 - 12.00 | 6.00 - 12.00 |  |
|  | **Rejected** | 0 | 0 | 0 |  |
| Right frontal | **FWHM (ppm)** |  |  |  | **0.035** |
|  | Mean (SD) | 0.048 (0.009) | 0.049 (0.008) | 0.054 (0.009) |  |
|  | Range | 0.033 - 0.076 | 0.038 - 0.076 | 0.038 - 0.067 |  |
|  | **SNR** |  |  |  | **0.021** |
|  | Mean (SD) | 16.28 (1.97) | 16.22 (2.49) | 14.54 (2.60) |  |
|  | Range | 13.00 - 19.00 | 12.00 - 20.00 | 9.00 - 20.00 |  |
|  | **CRLB** |  |  |  | 0.2 |
|  | Mean (SD) | 8.16 (1.14) | 8.13 (1.01) | 8.71 (1.30) |  |
|  | Range | 7.00 - 11.00 | 7.00 - 10.00 | 7.00 - 12.00 |  |
|  | **Rejected** | 0 | 1 | 0 |  |
| Left/Ipsi temporal | **FWHM (ppm)** |  |  |  | 0.8 |
|  | Mean (SD) | 0.061 (0.010) | 0.064 (0.010) | 0.064 (0.011) |  |
|  | Range | 0.038 - 0.076 | 0.048 - 0.086 | 0.048 - 0.086 |  |
|  | **SNR** |  |  |  | 0.4 |
|  | Mean (SD) | 12.00 (1.84) | 11.18 (1.65) | 11.38 (2.28) |  |
|  | Range | 9.00 - 16.00 | 8.00 - 14.00 | 8.00 - 17.00 |  |
|  | **CRLB** |  |  |  | 0.6 |
|  | Mean (SD) | 10.33 (1.99) | 10.55 (1.47) | 10.75 (1.89) |  |
|  | Range | 8.00 - 14.00 | 8.00 - 13.00 | 8.00 - 14.00 |  |
|  | **Rejected** | 1 | 2 | 4 |  |
| Right/Contra temporal | **FWHM (ppm)** |  |  |  | 0.8 |
|  | Mean (SD) | 0.059 (0.009) | 0.059 (0.014) | 0.060 (0.011) |  |
|  | Range | 0.043 - 0.076 | 0.038 - 0.086 | 0.038 - 0.076 |  |
|  | **SNR** |  |  |  | 0.2 |
|  | Mean (SD) | 11.83 (1.37) | 12.19 (2.09) | 11.42 (1.84) |  |
|  | Range | 9.00 - 14.00 | 8.00 - 16.00 | 8.00 - 15.00 |  |
|  | **CRLB** |  |  |  | >0.9 |
|  | Mean (SD) | 9.50 (1.56) | 9.52 (1.86) | 9.65 (1.77) |  |
|  | Range | 7.00 - 13.00 | 7.00 - 14.00 | 7.00 - 13.00 |  |
|  | **Rejected** | 1 | 3 | 2 |  |
| ^1^SD - standard deviation, FWHM (ppm) – full width at half maximum in parts per million, SNR – signal to noise ratio, CRLB – Cramér-Rao lower bound of relative standard deviation of estimated metabolite concentration, Ipsi – side ipsilateral to the perceived unilateral tinnitus, Contra – side contralateral to the perceived unilateral tinnitus | | | | | |
| ^2^C - control, TU - tinnitus unilateral, TB - tinnitus bilateral | | | | | |
| ^3^Kruskal-Wallis rank sum test | | | | | |

**Table S3.** Creatine CRLB values. Rejected values come from measurements with FWHM greater than 0.1 ppm.


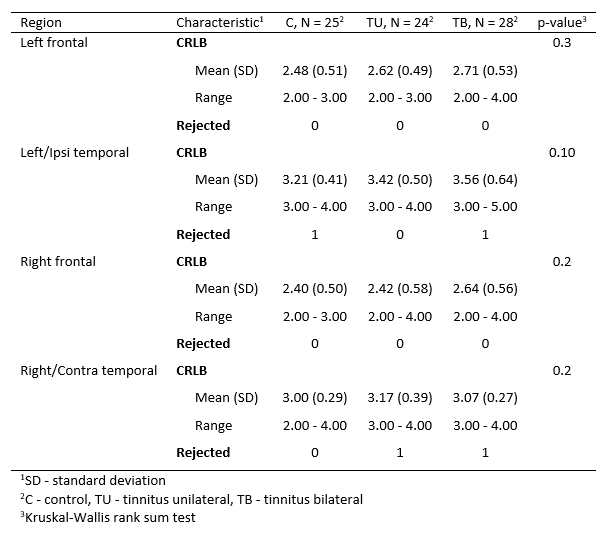


**
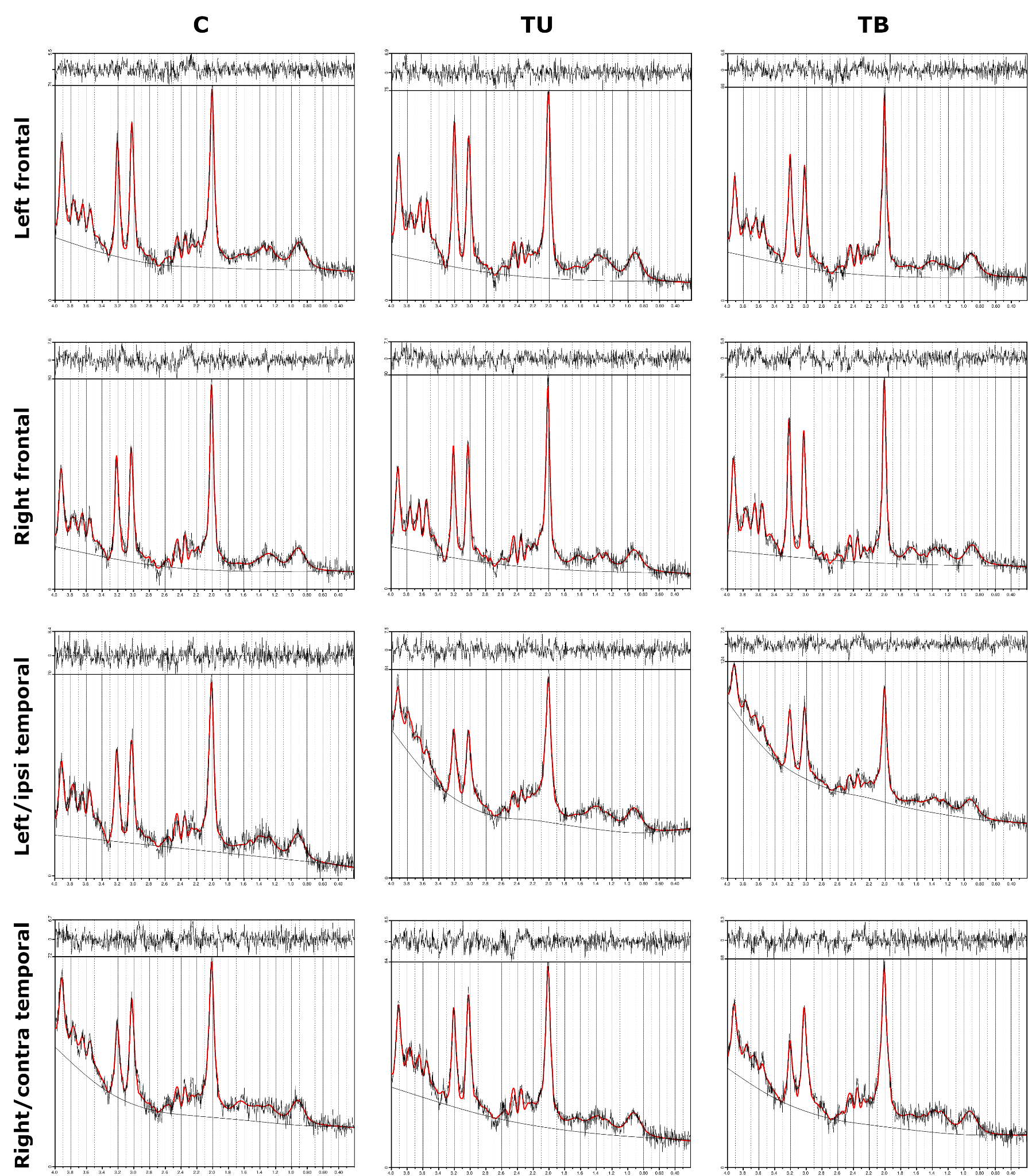
**

**Figure S3.** Representative spectra for each group and ROI (selected based on SNR and FWHM close to the average values in each group/voxel). Red lines represent models fitted by LCModel software, thin lines below spectra represent baseline, and the residuals are plotted above the spectra; C - control group, TU - unilateral tinnitus, TB - bilateral tinnitus.

**Table S4.** Brain tissue segmentation (GM and WM percentages) and between-group comparisons.

| Region^1^ | Tissue^1^ | **C**, N = 24^2^ | **TU**, N = 23^2^ | **TB**, N = 28^2^ | p-value^3^ |
| --- | --- | --- | --- | --- | --- |
| Left frontal | **GM** |  |  |  | 0.4 |
|  | Mean (SD) | 15.5 (5.8) | 17.9 (7.1) | 16.9 (6.1) |  |
|  | Range | 5.2 - 26.1 | 2.8 - 29.3 | 7.1 - 29.7 |  |
|  | **WM** |  |  |  | 0.5 |
|  | Mean (SD) | 84 (6) | 82 (7) | 83 (7) |  |
|  | Range | 73 - 95 | 70 - 97 | 68 - 93 |  |
| Right frontal | **GM** |  |  |  | 0.8 |
|  | Mean (SD) | 16 (8) | 17 (6) | 17 (7) |  |
|  | Range | 3 - 35 | 3 - 30 | 3 - 35 |  |
|  | **WM** |  |  |  | 0.8 |
|  | Mean (SD) | 83 (9) | 82 (7) | 83 (7) |  |
|  | Range | 61 - 97 | 69 - 97 | 65 - 97 |  |
| Left/Ipsi temporal | **GM** |  |  |  | 0.9 |
|  | Mean (SD) | 34 (8) | 36 (9) | 35 (7) |  |
|  | Range | 19 - 49 | 20 - 56 | 21 - 48 |  |
|  | **WM** |  |  |  | 0.8 |
|  | Mean (SD) | 64 (8) | 62 (10) | 64 (7) |  |
|  | Range | 50 - 80 | 43 - 80 | 51 - 79 |  |
| Right/Contra temporal | **GM** |  |  |  | 0.6 |
|  | Mean (SD) | 44 (7) | 43 (6) | 45 (6) |  |
|  | Range | 31 - 58 | 33 - 55 | 33 - 60 |  |
|  | **WM** |  |  |  | 0.6 |
|  | Mean (SD) | 53 (7) | 54 (6) | 52 (7) |  |
|  | Range | 38 - 64 | 43 - 63 | 38 - 65 |  |
| ^1^SD - standard deviation, Ipsi – side ipsilateral to the perceived unilateral tinnitus, Contra – side contralateral to the perceived unilateral tinnitus | | | | | |
| ^2^C - control, TU - tinnitus unilateral, TB - tinnitus bilateral | | | | | |
| ^3^Kruskal-Wallis rank sum test | | | | | |

**Table S5.** Other metabolite levels: ml/tCr, tCho/tCr and tNAA/tCr CRLB.

| Region^1^ | Metabolite | Characteristic^1^ | C, N = 25^2^ | TU, N = 24^2^ | TB, N = 28^2^ | p-value^3^ |
| --- | --- | --- | --- | --- | --- | --- |
| Left frontal | mI | **CRLB** |  |  |  | 0.2 |
|  |  | Mean (SD) | 5.96 (0.98) | 6.12 (1.33) | 6.54 (1.10) |  |
|  |  | Range | 4.00 - 8.00 | 4.00 - 9.00 | 5.00 - 9.00 |  |
|  |  | **Rejected** | 0 | 0 | 0 |  |
|  | tCho | **CRLB** |  |  |  | 0.7 |
|  |  | Mean (SD) | 2.92 (0.57) | 2.79 (0.41) | 2.82 (0.48) |  |
|  |  | Range | 2.00 - 4.00 | 2.00 - 3.00 | 2.00 - 4.00 |  |
|  |  | **Rejected** | 0 | 0 | 0 |  |
|  | tNAA | **CRLB** |  |  |  | 0.11 |
|  |  | Mean (SD) | 2.76 (0.44) | 2.83 (0.56) | 3.07 (0.60) |  |
|  |  | Range | 2.00 - 3.00 | 2.00 - 4.00 | 2.00 - 4.00 |  |
|  |  | **Rejected** | 0 | 0 | 0 |  |
| Right frontal | mI | **CRLB** |  |  |  | 0.2 |
|  |  | Mean (SD) | 6.28 (1.37) | 6.04 (1.40) | 6.64 (1.13) |  |
|  |  | Range | 4.00 - 10.00 | 4.00 - 9.00 | 5.00 - 9.00 |  |
|  |  | **Rejected** | 0 | 1 | 0 |  |
|  | tCho | **CRLB** |  |  |  | 0.12 |
|  |  | Mean (SD) | 2.84 (0.47) | 2.67 (0.64) | 2.96 (0.51) |  |
|  |  | Range | 2.00 - 4.00 | 2.00 - 4.00 | 2.00 - 4.00 |  |
|  |  | **Rejected** | 0 | 0 | 0 |  |
|  | tNAA | **CRLB** |  |  |  | 0.7 |
|  |  | Mean (SD) | 2.80 (0.58) | 2.79 (0.72) | 2.89 (0.63) |  |
|  |  | Range | 2.00 - 4.00 | 2.00 - 5.00 | 2.00 - 4.00 |  |
|  |  | **Rejected** | 0 | 0 | 0 |  |
| Left/Ipsi temporal | mI | **CRLB** |  |  |  | 0.8 |
|  |  | Mean (SD) | 8.61 (2.06) | 9.09 (2.65) | 9.09 (2.29) |  |
|  |  | Range | 6.00 - 14.00 | 6.00 - 14.00 | 6.00 - 15.00 |  |
|  |  | **Rejected** | 2 | 2 | 6 |  |
|  | tCho | **CRLB** |  |  |  | 0.3 |
|  |  | Mean (SD) | 3.75 (0.68) | 4.12 (0.80) | 4.00 (0.96) |  |
|  |  | Range | 3.00 - 5.00 | 3.00 - 6.00 | 3.00 - 7.00 |  |
|  |  | **Rejected** | 1 | 0 | 1 |  |
|  | tNAA | **CRLB** |  |  |  | 0.3 |
|  |  | Mean (SD) | 3.62 (0.65) | 3.96 (0.69) | 3.93 (1.04) |  |
|  |  | Range | 3.00 - 5.00 | 3.00 - 5.00 | 3.00 - 7.00 |  |
|  |  | **Rejected** | 1 | 0 | 1 |  |
| Right/Contra temporal | mI | **CRLB** |  |  |  | 0.5 |
|  |  | Mean (SD) | 8.95 (1.99) | 8.52 (1.81) | 9.16 (2.10) |  |
|  |  | Range | 6.00 - 15.00 | 6.00 - 14.00 | 6.00 - 14.00 |  |
|  |  | **Rejected** | 3 | 3 | 3 |  |
|  | tCho | **CRLB** |  |  |  | 0.9 |
|  |  | Mean (SD) | 3.96 (0.73) | 4.04 (0.82) | 3.96 (0.85) |  |
|  |  | Range | 3.00 - 6.00 | 3.00 - 6.00 | 3.00 - 7.00 |  |
|  |  | **Rejected** | 0 | 1 | 1 |  |
|  | tNAA | **CRLB** |  |  |  | 0.3 |
|  |  | Mean (SD) | 3.76 (0.60) | 3.91 (0.90) | 4.04 (0.65) |  |
|  |  | Range | 3.00 - 5.00 | 3.00 - 6.00 | 3.00 - 5.00 |  |
|  |  | **Rejected** | 0 | 1 | 1 |  |
| ^1^SD - standard deviation, Ipsi – side ipsilateral to the perceived unilateral tinnitus, Contra – side contralateral to the perceived unilateral tinnitus | | | | | | |
| ^2^C - control, TU - tinnitus unilateral, TB - tinnitus bilateral | | | | | | |
| ^3^Kruskal-Wallis rank sum test | | | | | | |


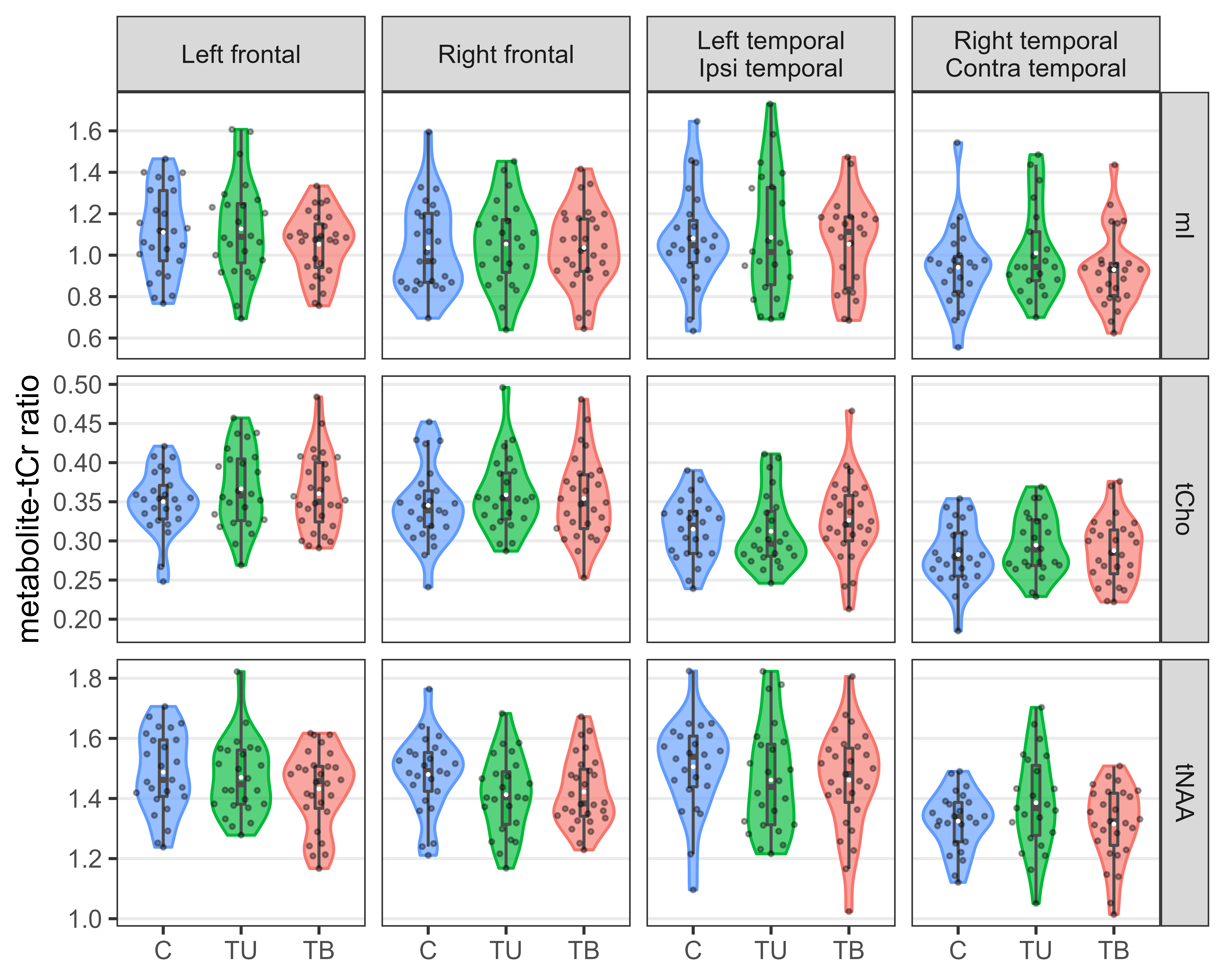


**Figure S4.** tNAA/tCr, tCho/tCr and mI/tCr levels in the four ROIs. Individual subjects are depicted as points in the violin plots. White dots represent group means, while middle horizontal bars on box plots represent medians.  TB - bilateral tinnitus, TU - unilateral tinnitus, C - control;  Ipsi - the side corresponding to the side of tinnitus in the TU group (for 4 TU participants that were experiencing tinnitus on the right side, the sides were switched), Contra – side opposite to tinnitus laterality.

**Table S6.** tNAA/tCr levels in four regions of interest compared across the groups.

|  | Descriptive statistics | | | Welch’s ANOVA | | | | Post-hoc tests | | |
| --- | --- | --- | --- | --- | --- | --- | --- | --- | --- | --- |
| Region^1^ | C, N = 25^2^ | TU, N = 24^2^ | TB, N = 28^2^ | F | p | df1 | df2 | C vs. TU^3^ | C vs. TB^3^ | TU vs. TB^3^ |
| **Left frontal** |  |  |  | 1.2 | 0.3 | 2.00 | 48.9 | > 0.9 | 0.4 | 0.9 |
| Median (IQR) | 1.46 (1.41 - 1.60) | 1.45 (1.38 - 1.56) | 1.46 (1.37 - 1.51) |  |  |  |  |  |  |  |
| Mean (SD) | 1.49 (0.13) | 1.47 (0.13) | 1.43 (0.13) |  |  |  |  |  |  |  |
| Range | 1.24 - 1.71 | 1.28 - 1.82 | 1.17 - 1.62 |  |  |  |  |  |  |  |
| S-W test | 0.48 | 0.22 | 0.10 |  |  |  |  |  |  |  |
| Missing | 0 | 0 | 0 |  |  |  |  |  |  |  |
| **Right frontal** |  |  |  | 2.1 | 0.14 | 2.00 | 48.2 | 0.2 | 0.3 | > 0.9 |
| Median (IQR) | 1.49 (1.42 - 1.55) | 1.40 (1.31 - 1.49) | 1.38 (1.34 - 1.50) |  |  |  |  |  |  |  |
| Mean (SD) | 1.48 (0.13) | 1.41 (0.13) | 1.42 (0.12) |  |  |  |  |  |  |  |
| Range | 1.21 - 1.76 | 1.17 - 1.68 | 1.23 - 1.67 |  |  |  |  |  |  |  |
| S-W test | 0.37 | 0.95 | 0.36 |  |  |  |  |  |  |  |
| Missing | 0 | 0 | 0 |  |  |  |  |  |  |  |
| **Left/Ipsi temporal** |  |  |  | 0.61 | 0.5 | 2.00 | 47.6 | > 0.9 | > 0.9 | > 0.9 |
| Median (IQR) | 1.54 (1.44 - 1.61) | 1.44 (1.31 - 1.58) | 1.48 (1.39 - 1.57) |  |  |  |  |  |  |  |
| Mean (SD) | 1.51 (0.15) | 1.46 (0.18) | 1.46 (0.17) |  |  |  |  |  |  |  |
| Range | 1.10 - 1.82 | 1.22 - 1.82 | 1.02 - 1.81 |  |  |  |  |  |  |  |
| S-W test | 0.23 | 0.20 | 0.75 |  |  |  |  |  |  |  |
| Missing | 1 | 0 | 1 |  |  |  |  |  |  |  |
| **Right/Contra temporal** |  |  |  | 1.6 | 0.2 | 2.00 | 45.1 | 0.4 | > 0.9 | 0.3 |
| Median (IQR) | 1.34 (1.26 - 1.39) | 1.37 (1.28 - 1.51) | 1.32 (1.24 - 1.42) |  |  |  |  |  |  |  |
| Mean (SD) | 1.32 (0.10) | 1.39 (0.16) | 1.32 (0.13) |  |  |  |  |  |  |  |
| Range | 1.12 - 1.49 | 1.05 - 1.70 | 1.01 - 1.51 |  |  |  |  |  |  |  |
| S-W test | 0.62 | 1.00 | 0.21 |  |  |  |  |  |  |  |
| Missing | 0 | 1 | 1 |  |  |  |  |  |  |  |
| ^1^SD - standard deviation, IQR - interquartile interval, S-W test - Shapiro-Wilk test of normality result (p-value), Ipsi – side ipsilateral to the perceived unilateral tinnitus, Contra – side contralateral to the perceived unilateral tinnitus | | | | | | | | | | |
| ^2^C - control, TU - tinnitus unilateral, TB - tinnitus bilateral | | | | | | | | | | |
| ^3^Welch t test; Bonferroni correction | | | | | | | | | | |

**Table S7.** tCho/tCr levels in four regions of interest compared across the groups.

|  | Descriptive statistics | | | Welch’s ANOVA | | | | Post-hoc tests | | |
| --- | --- | --- | --- | --- | --- | --- | --- | --- | --- | --- |
| Region^1^ | C, N = 25^2^ | TU, N = 24^2^ | TB, N = 28^2^ | F | p | df1 | df2 | C vs. TU^3^ | C vs. TB^3^ | TU vs. TB^3^ |
| **Left frontal** |  |  |  | 0.82 | 0.4 | 2.00 | 48.5 | 0.7 | > 0.9 | > 0.9 |
| Median (IQR) | 0.35 (0.33 - 0.37) | 0.36 (0.33 - 0.40) | 0.35 (0.32 - 0.40) |  |  |  |  |  |  |  |
| Mean (SD) | 0.35 (0.04) | 0.37 (0.05) | 0.36 (0.05) |  |  |  |  |  |  |  |
| Range | 0.25 - 0.42 | 0.27 - 0.46 | 0.29 - 0.48 |  |  |  |  |  |  |  |
| S-W test | 0.34 | 0.69 | 0.24 |  |  |  |  |  |  |  |
| Missing | 0 | 0 | 0 |  |  |  |  |  |  |  |
| **Right frontal** |  |  |  | 0.47 | 0.6 | 2.00 | 49.1 | > 0.9 | > 0.9 | > 0.9 |
| Median (IQR) | 0.34 (0.32 - 0.36) | 0.35 (0.33 - 0.39) | 0.35 (0.32 - 0.38) |  |  |  |  |  |  |  |
| Mean (SD) | 0.35 (0.05) | 0.36 (0.05) | 0.35 (0.05) |  |  |  |  |  |  |  |
| Range | 0.24 - 0.45 | 0.29 - 0.50 | 0.25 - 0.48 |  |  |  |  |  |  |  |
| S-W test | 0.35 | 0.16 | 0.69 |  |  |  |  |  |  |  |
| Missing | 0 | 0 | 0 |  |  |  |  |  |  |  |
| **Left/Ipsi temporal** |  |  |  | 0.57 | 0.6 | 2.00 | 47.7 | > 0.9 | > 0.9 | > 0.9 |
| Median (IQR) | 0.32 (0.28 - 0.34) | 0.30 (0.28 - 0.34) | 0.32 (0.30 - 0.36) |  |  |  |  |  |  |  |
| Mean (SD) | 0.32 (0.04) | 0.31 (0.05) | 0.33 (0.05) |  |  |  |  |  |  |  |
| Range | 0.24 - 0.39 | 0.25 - 0.41 | 0.21 - 0.47 |  |  |  |  |  |  |  |
| S-W test | 0.95 | 0.02 | 0.75 |  |  |  |  |  |  |  |
| Missing | 1 | 0 | 1 |  |  |  |  |  |  |  |
| **Right/Contra temporal** |  |  |  | 0.56 | 0.6 | 2.00 | 47.7 | 0.9 | > 0.9 | > 0.9 |
| Median (IQR) | 0.28 (0.26 - 0.31) | 0.29 (0.27 - 0.33) | 0.29 (0.26 - 0.31) |  |  |  |  |  |  |  |
| Mean (SD) | 0.28 (0.04) | 0.29 (0.04) | 0.29 (0.04) |  |  |  |  |  |  |  |
| Range | 0.19 - 0.35 | 0.23 - 0.37 | 0.22 - 0.38 |  |  |  |  |  |  |  |
| S-W test | 0.48 | 0.35 | 0.54 |  |  |  |  |  |  |  |
| Missing | 0 | 1 | 1 |  |  |  |  |  |  |  |
| ^1^SD - standard deviation, IQR - interquartile interval, S-W test - Shapiro-Wilk test of normality result (p-value), Ipsi – side ipsilateral to the perceived unilateral tinnitus, Contra – side contralateral to the perceived unilateral tinnitus | | | | | | | | | | |
| ^2^C - control, TU - tinnitus unilateral, TB - tinnitus bilateral | | | | | | | | | | |
| ^3^Welch t test; Bonferroni correction | | | | | | | | | | |

**Table S8.** mI/tCr levels in four regions of interest compared across the groups.

|  | Descriptive statistics | | | Welch’s ANOVA | | | | Post-hoc tests | | |
| --- | --- | --- | --- | --- | --- | --- | --- | --- | --- | --- |
| Region^1^ | C, N = 25^2^ | TU, N = 24^2^ | TB, N = 28^2^ | F | p | df1 | df2 | C vs. TU^3^ | C vs. TB^3^ | TU vs. TB^3^ |
| **Left frontal** |  |  |  | 1.2 | 0.3 | 2.00 | 45.6 | > 0.9 | 0.8 | 0.6 |
| Median (IQR) | 1.12 (0.97 - 1.31) | 1.09 (0.96 - 1.25) | 1.08 (0.94 - 1.15) |  |  |  |  |  |  |  |
| Mean (SD) | 1.11 (0.21) | 1.13 (0.24) | 1.05 (0.15) |  |  |  |  |  |  |  |
| Range | 0.77 - 1.47 | 0.69 - 1.61 | 0.76 - 1.33 |  |  |  |  |  |  |  |
| S-W test | 0.36 | 0.66 | 0.42 |  |  |  |  |  |  |  |
| Missing | 0 | 0 | 0 |  |  |  |  |  |  |  |
| **Right frontal** |  |  |  | 0.06 | >0.9 | 2.00 | 47.3 | > 0.9 | > 0.9 | > 0.9 |
| Median (IQR) | 0.97 (0.87 - 1.20) | 1.06 (0.92 - 1.17) | 1.03 (0.92 - 1.17) |  |  |  |  |  |  |  |
| Mean (SD) | 1.04 (0.21) | 1.05 (0.21) | 1.04 (0.19) |  |  |  |  |  |  |  |
| Range | 0.70 - 1.60 | 0.64 - 1.45 | 0.65 - 1.42 |  |  |  |  |  |  |  |
| S-W test | 0.06 | 0.99 | 0.84 |  |  |  |  |  |  |  |
| Missing | 0 | 1 | 0 |  |  |  |  |  |  |  |
| **Left/Ipsi temporal** |  |  |  | 0.12 | 0.9 | 2.00 | 41.9 | > 0.9 | > 0.9 | > 0.9 |
| Median (IQR) | 1.05 (0.96 - 1.17) | 1.02 (0.86 - 1.33) | 1.11 (0.84 - 1.18) |  |  |  |  |  |  |  |
| Mean (SD) | 1.08 (0.24) | 1.09 (0.30) | 1.05 (0.22) |  |  |  |  |  |  |  |
| Range | 0.63 - 1.65 | 0.69 - 1.73 | 0.69 - 1.47 |  |  |  |  |  |  |  |
| S-W test | 0.59 | 0.19 | 0.17 |  |  |  |  |  |  |  |
| Missing | 2 | 2 | 6 |  |  |  |  |  |  |  |
| **Right/Contra temporal** |  |  |  | 0.93 | 0.4 | 2.00 | 42.0 | 0.9 | > 0.9 | 0.6 |
| Median (IQR) | 0.95 (0.83 - 0.99) | 0.94 (0.88 - 1.11) | 0.93 (0.81 - 0.96) |  |  |  |  |  |  |  |
| Mean (SD) | 0.94 (0.20) | 1.01 (0.22) | 0.93 (0.19) |  |  |  |  |  |  |  |
| Range | 0.56 - 1.54 | 0.70 - 1.49 | 0.62 - 1.44 |  |  |  |  |  |  |  |
| S-W test | 0.12 | 0.03 | 0.10 |  |  |  |  |  |  |  |
| Missing | 3 | 3 | 3 |  |  |  |  |  |  |  |
| ^1^SD - standard deviation, IQR - interquartile interval, S-W test - Shapiro-Wilk test of normality result (p-value), Ipsi – side ipsilateral to the perceived unilateral tinnitus, Contra – side contralateral to the perceived unilateral tinnitus | | | | | | | | | | |
| ^2^C - control, TU - tinnitus unilateral, TB - tinnitus bilateral | | | | | | | | | | |
| ^3^Welch t test; Bonferroni correction | | | | | | | | | | |

**
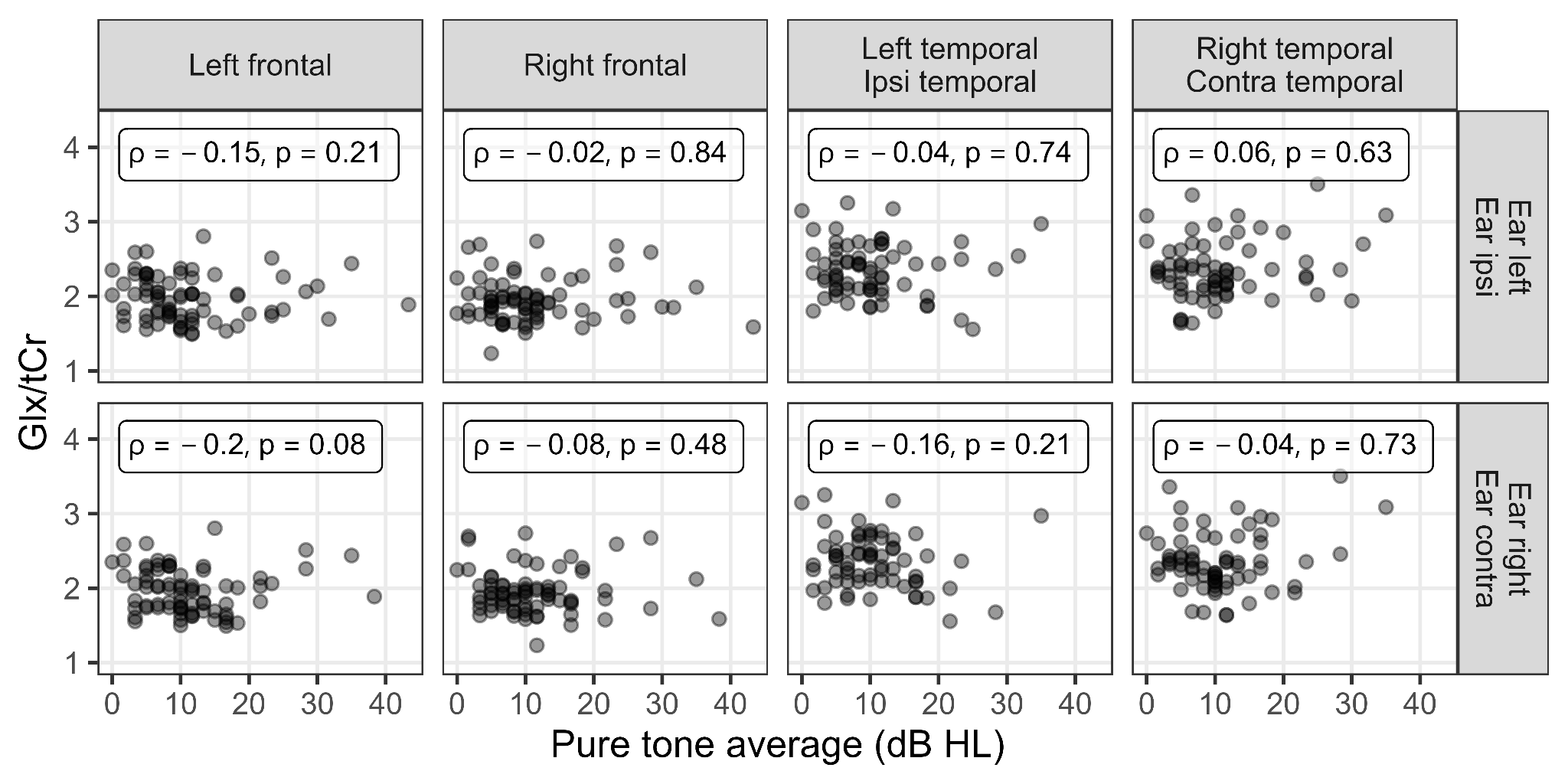
**

**Figure S5.** Scatterplots and Spearman’s ρ tests for correlation between PTA values (500-2000 Hz) in both ears separately and levels of Glx/tCr in each ROI. The analysis was performed on data points conforming with the quality criteria. Ipsi – side ipsilateral to the perceived unilateral tinnitus, Contra – side contralateral to the perceived unilateral tinnitus.


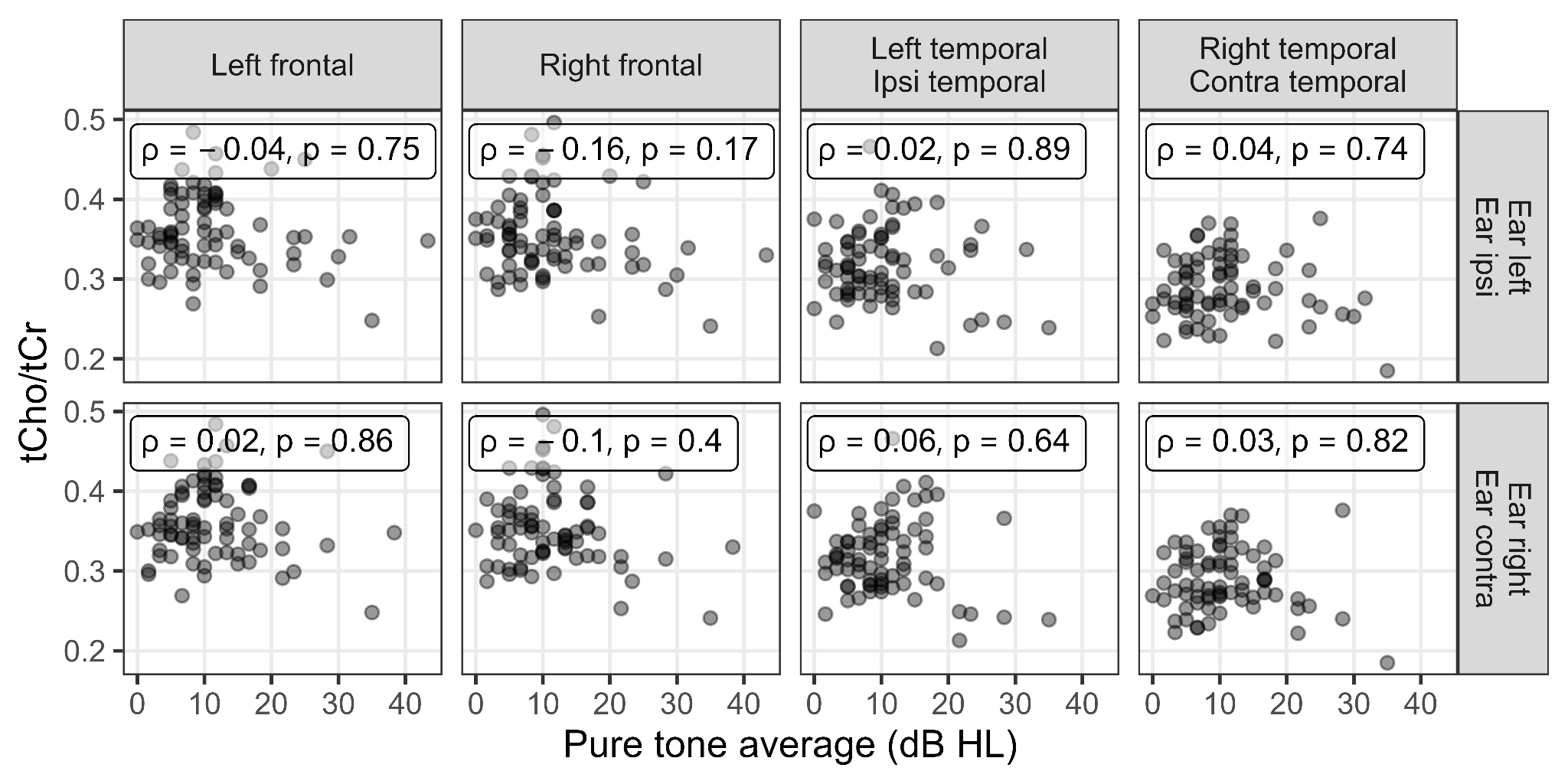


**Figure S6.** Scatterplots and Spearman’s ρ tests for correlation between PTA values (500-2000 Hz) in both ears separately and levels of tCho/tCr in four ROIs. The analysis was performed on data points conforming with the quality criteria. Ipsi – side ipsilateral to the perceived unilateral tinnitus, Contra – side contralateral to the perceived unilateral tinnitus.

**
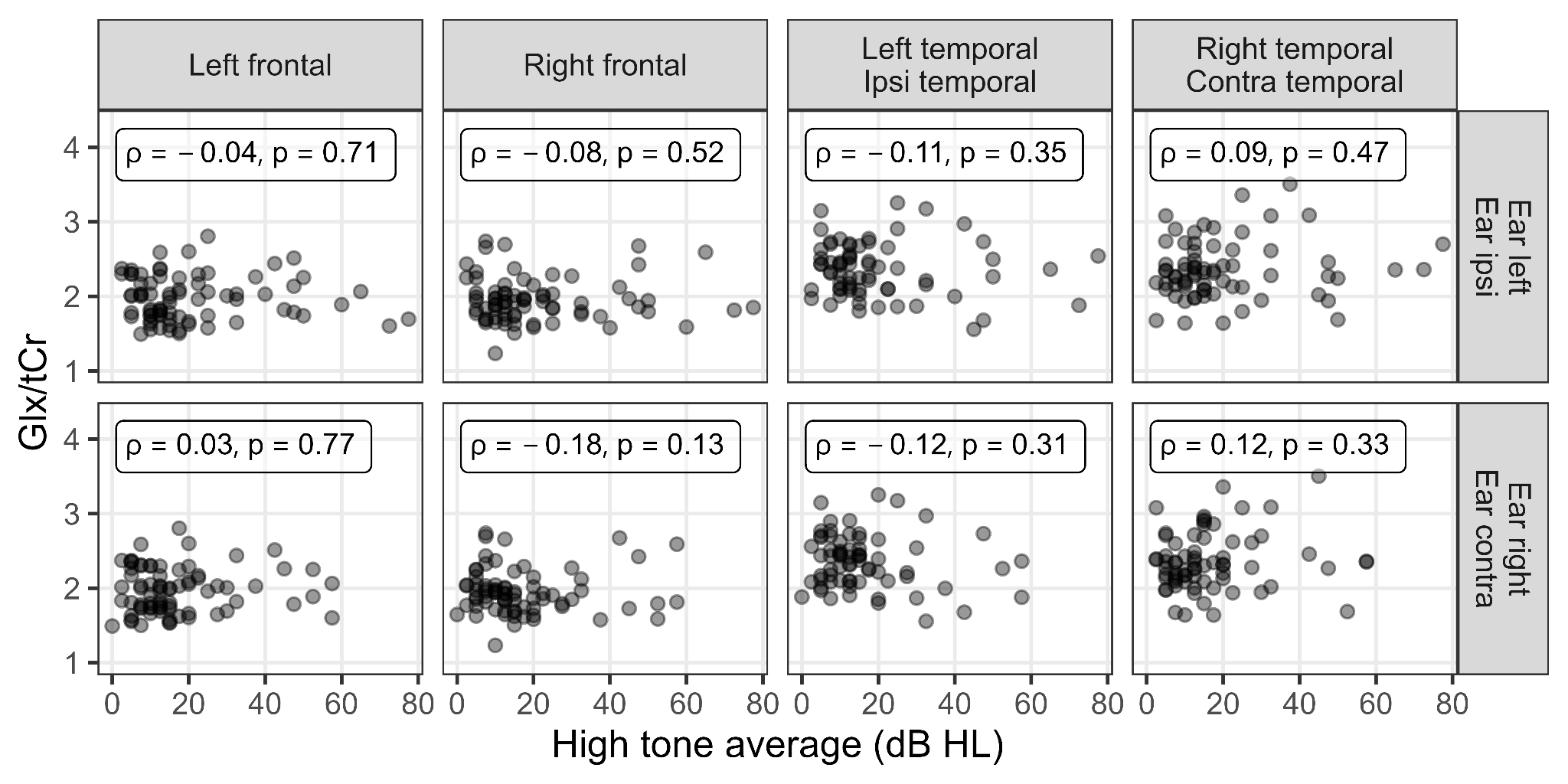
**

**Figure S7.** Scatterplots and Spearman’s ρ tests for correlation between HTA values (4000-8000 Hz) in both ears separately and levels of Glx/tCr in each ROI. The analysis was performed on data points conforming with the quality criteria. Ipsi – side ipsilateral to the perceived unilateral tinnitus, Contra – side contralateral to the perceived unilateral tinnitus.


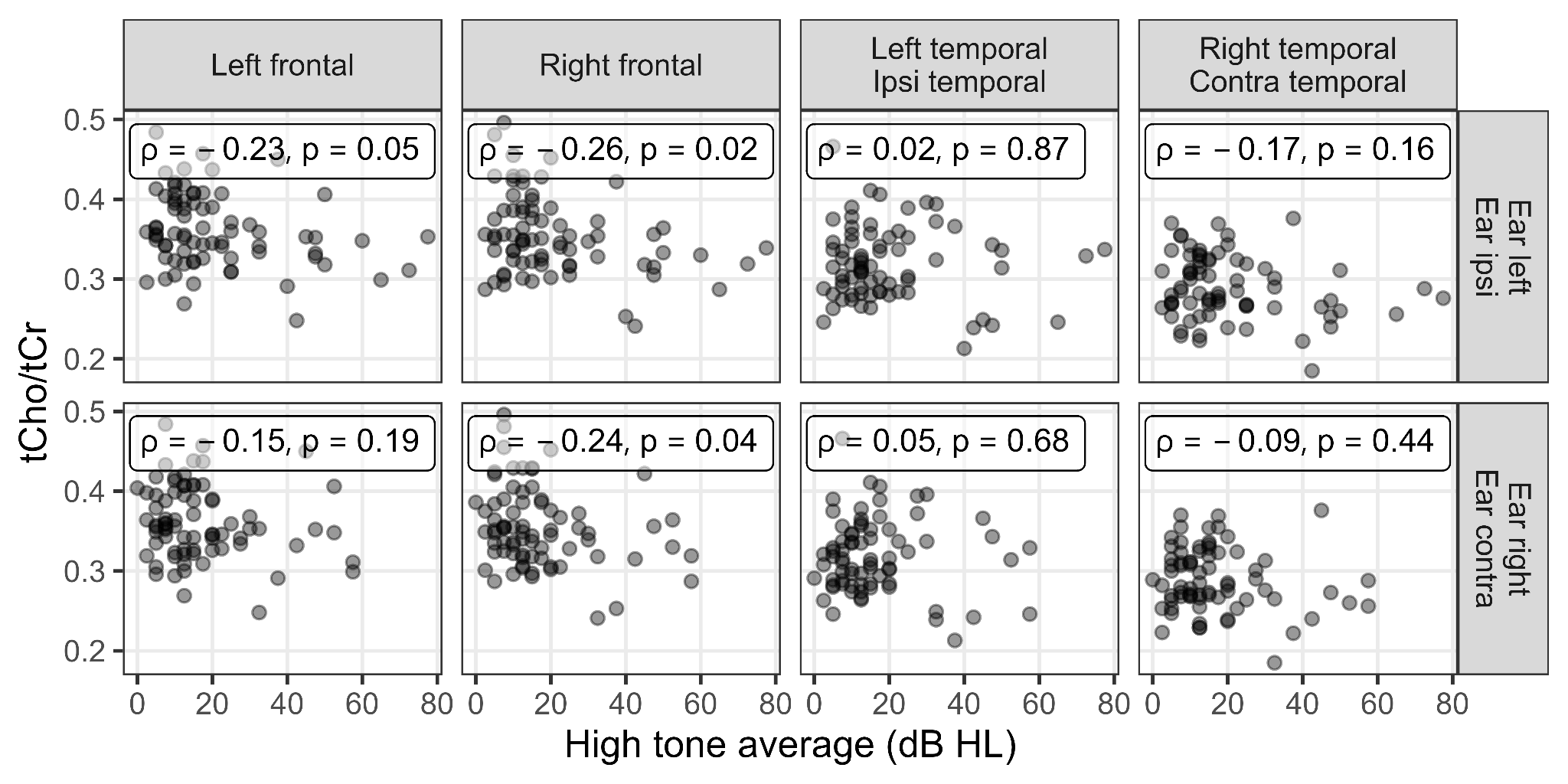


**Figure S8.** Scatterplots and Spearman’s ρ tests for correlation between HTA values (4000-8000 Hz) in both ears separately and levels of tCho/tCr in four ROIs. The analysis was performed on data points conforming with the quality criteria. Ipsi – side ipsilateral to the perceived unilateral tinnitus, Contra – side contralateral to the perceived unilateral tinnitus.

**Supplementary File 1.** (File_1_LF.nii) Group coverage mask for left frontal ROI.

**Supplementary File 2.** (File_2_RF.nii) Group coverage mask for right frontal ROI.

**Supplementary File 3.** (File_3_LT.nii) Group coverage mask for left temporal ROI.

**Supplementary File 4.** (File_4_RT.nii) Group coverage mask for right temporal ROI.
